# Cancer Stem Cell Subpopulations Are Present Within Metastatic Head and Neck Cutaneous Squamous Cell Carcinoma

**DOI:** 10.3389/fonc.2020.01091

**Published:** 2020-07-30

**Authors:** Ethan J. Kilmister, Josie Patel, Bede van Schaijik, Nicholas Bockett, Helen D. Brasch, Erin Paterson, Dalice Sim, Paul F. Davis, Imogen M. Roth, Tinte Itinteang, Swee T. Tan

**Affiliations:** ^1^Gillies McIndoe Research Institute, Wellington, New Zealand; ^2^Biostatistical Group/Dean's Department, University of Otago, Wellington, New Zealand; ^3^Wellington Regional Plastic, Maxillofacial and Burns Unit, Hutt Hospital, Wellington, New Zealand; ^4^Department of Surgery, The University of Melbourne, Parkville, VIC, Australia

**Keywords:** metastatic cutaneous squamous cell carcinoma, cancer stem cells, induced pluripotent stem cells, embryonic stem cells, head and neck cancer, nodal metastasis

## Abstract

Cancer stem cells (CSCs) have been identified in many cancer types including primary head and neck cutaneous squamous cell carcinoma (HNcSCC). This study aimed to identify and characterize CSCs in metastatic HNcSCC (mHNcSCC). Immunohistochemical staining performed on mHNcSCC samples from 15 patients demonstrated expression of the induced pluripotent stem cell (iPSC) markers OCT4, SOX2, NANOG, KLF4, and c-MYC in all 15 samples. *In situ* hybridization and RT-qPCR performed on four of these mHNcSCC tissue samples confirmed transcript expression of all five iPSC markers. Immunofluorescence staining performed on three of these mHNcSCC samples demonstrated expression of c-MYC on cells within the tumor nests (TNs) and the peri-tumoral stroma (PTS) that also expressed KLF4. OCT4 was expressed on the SOX2+/NANOG+/KLF4+ cells within the TNs, and the SOX2+/NANOG+/KLF4+ cells within the PTS. RT-qPCR demonstrated transcript expression of all five iPSC markers in all three mHNcSCC-derived primary cell lines, except for SOX2 in one cell line. Western blotting showed the presence of SOX2, KLF4, and c-MYC but not OCT4 and NANOG in the three mHNcSCC-derived primary cell lines. All three cell lines formed tumorspheres, at the first passage. We demonstrated an OCT4+/NANOG+/SOX2+/KLF4+/c-MYC+ CSC subpopulation and an OCT4+/NANOG-/SOX2+/KLF4+/c-MYC+ subpopulation within the TNs, and an OCT4+/NANOG+/SOX2+/KLF4+/c-MYC+ subpopulation within the PTS of mHNcSCC.

## Introduction

The incidence of non-melanoma skin cancer (NMSC) is rising rapidly globally, particularly amongst Anglo-Celtic populations (1). Cutaneous squamous cell carcinoma (cSCC) is the second most common form of NMSC, affecting 118/100,000 people in New Zealand (1). Caucasian descent, pale complexion and advancing age increase the risk of developing cSCC (2). Tumor thickness, horizontal diameter, perineural and/or lymphovascular invasion, poor histological differentiation and certain anatomic sites increase the metastatic risk (1, 3–5).

Sixty percent of cSCC occur in the head and neck with a 2% incidence of metastasis, mainly to the parotid and/or neck nodes (4), making metastatic cSCC the most common parotid malignancy in New Zealand and Australia (6). Treatment for metastatic head and neck cSCC (mHNcSCC) is surgery and post-operative adjuvant radiotherapy with a 48% 5-year overall survival (2). This poor outcome has been attributed to the presence of cancer stem cells (CSCs) (7).

The CSC concept proposes that the development and progression of cancer are driven by CSCs (8), a small population of highly tumorigenic cells imbued with embryonic stem cell (ESC) properties (9), such as self-renewal (10), and pluripotency (9). CSCs divide asymmetrically to produce identical CSCs, as well as differentiated cancer cells that possess little or no tumorigenicity and form the bulk of the tumor (11, 12).

Yamanaka et al. (13, 14) have demonstrated induction of pluripotent stem cells from adult mouse and human fibroblasts by the introduction of the ESC markers OCT4, SOX2, c-MYC, and KLF4. Thomson et al. (15) have demonstrated that this can also be achieved with NANOG and LIN28 in place of c-MYC and KLF4. These studies underscore the sufficiency of these four transcription factors (13) to generate induced pluripotent stem cells (iPSCs). CSCs in several cancer types (16–18) including primary head and neck cSCC (HNcSCC) (19) have been shown to express these master regulators of pluripotency.

SOX2, a member of the SRY-related HMG-box (SOX) gene family of transcription factors that maintains developmental potential (20), is a marker of CSCs in cSCC (21). SOX2 expression is critical for tumor initiation and growth, and regulates self-renewal and long-term growth of CSCs (21–23). OCT4, a POU domain transcription factor (24), forms the “core pluripotency network” with SOX2 and NANOG to regulate ESC pluripotency (25) and stemness of CSCs in HNcSCC (26). Its expression correlates with poor overall survival of patients with oral cavity SCC (OCSCC) (27). NANOG, a homeodomain containing transcription factor (28), also correlates with worsened survival of OCSCC (27). NANOG induces CSC characteristics in OCSCC (27) and colorectal cancer (29). KLF4, a zinc-finger transcription factor and part of the Krüppel-like family, plays a central role in cell cycle regulation, maintenance of pluripotency and somatic cell reprogramming (30). c-MYC, an oncoprotein within the MYC family, is involved in cell growth, differentiation, apoptosis, angiogenesis, and stem cell pluripotency and proliferation (31, 32). MYC dysregulation occurs in various cancer types (33–35) and is associated with more aggressive tumors (36).

OCT4, SOX2, NANOG, KLF4, and c-MYC act cooperatively to promote pluripotency (37), and consequently, some or all of these markers have been used to identify CSC subpopulations in many cancer types, including HNcSCC (19), OCSCC affecting different subsites (38–40), glioblastoma (41), renal clear cell carcinoma (17), primary (18), and metastatic (42) colon adenocarcinoma, and metastatic malignant melanoma (43, 44).

We have recently demonstrated the presence of CSCs in primary HNcSCC that express the iPSC markers OCT4, SOX2, NANOG, KLF4, and c-MYC (19). This study aimed to identify and characterize CSCs within mHNcSCC using these markers.

## Materials and Methods

### mHNcSCC Tissue Samples

Formalin-fixed paraffin-embedded (FFPE) sections of mHNcSCC tissue samples from 15 male patients, aged 53–92 (mean, 80) years (Table 1) were sourced from the Gillies McIndoe Research Institute Tissue Bank for this study, which was approved by the Central Regional Health and Disability Ethics Committee (Ref. 12/CEN/74AM05). The mHNcSCC tissue samples used were from a separate cohort of patients than those used in our previous study that investigated CSCs in primary HNcSCC (19). Written informed consent was obtained from all patients.

**Table 1:** Patient demographics and site of nodal metastasis of metastatic cutaneous head and neck squamous cell carcinoma.

**Patient**	**Gender**	**Age (years)**	**Site of nodal metastasis**
1	M	69	Neck
2	M	84	Parotid and neck
3	M	92	Parotid
4	M	90	Parotid
5	M	89	Parotid and neck
6	M	83	Parotid
7	M	89	Neck
8	M	82	Parotid and neck
9	M	53	Neck
10	M	74	Parotid and neck
11	M	74	Neck
12	M	85	Neck
13	M	78	Parotid
14	M	85	Neck
15	M	78	Parotid and neck

### mHNcSCC-Derived Primary Cell Lines

mHNcSCC-derived primary cell lines were established from three freshly excised mHNcSCC tissue samples from the 15 patients included in the IHC staining, and cultured as explants by placing them between layers of Matrigel (cat#354234, Corning Life Sciences, Tewksbury, MA, USA) in 24-well plates and adding an explant culture media DMEM (cat#10569010, Gibco, Rockford, IL, USA) + 2% penicillin-streptomycin (cat#15140122, Gibco) + 0.2% gentamycin/amphotericin B (cat#R01510, Gibco). Cells were extracted from the Matrigel following abundant growth using Dispase (cat#354235, Corning Life Sciences). The extracted cells were cultured and passaged in a cell culture media consisting of DMEM (1X) (Gibco) and GlutaMAX-1 (cat#10569-010, Life Technologies), DMEM medium supplemented with 10% fetal bovine serum (cat#10091148, Gibco), 5% mTeSR™ (cat#85850, StemCell Technologies, Vancouver, BC, Canada), 1% penicillin-streptomycin (Gibco) and 0.2% gentamicin/amphotericin B (Gibco). All cultures were maintained in a humidified incubator at 37°C with 5% CO_2_. All mHNcSCC-derived primary cell lines used for the experiments were at passages 4–8 (WB and RT-qPCR) or 7–9 (tumorsphere formation assays).

### Histochemical, Immunohistochemical, and Immunofluorescence Staining

Four micro meter-thick FFPE consecutive sections of mHNcSCC tissue samples from 15 patients underwent hematoxylin and eosin (H&E) staining to confirm the presence of mHNcSCC on the slides by an anatomical pathologist, and immunohistochemical IHC staining with the primary antibodies OCT4 (1:30; cat#MRQ-10, Cell Marque), SOX2 (1:500; cat#PA1-094, Thermo Fisher Scientific), NANOG (1:200; cat#EP225, Cell Marque), c-MYC (1:1000; cat#ab32, Abcam, Cambridge, MA, USA), and KLF4 (1:100; cat#NBP2-24749, Novus Biologicals, Littleton, CO, USA), with 3,3′-diaminobenzidine as the chromogen. All antibodies were diluted with BOND™ primary antibody diluent (cat#AR9352, Leica). IHC-stained slides were mounted in Surgipath Micromount mounting medium (cat#38017322, Leica).

Three mHNcSCC tissue samples from the original cohort of 15 patients underwent immunofluorescence (IF) staining to determine co-expression of proteins. VectaFluor Excel anti-mouse 488 (ready-to-use; cat#VEDK2488, Vector Laboratories, Burlingame, CA, USA) and Alexa Fluor anti-rabbit 594 (1:500; cat#A21207, Life Technologies, Carlsbad, CA, USA) were used to determine marker expression. All IF-stained slides were mounted in Vecta Shield Hardset mounting medium with 4′,6-diamidino-2-phenylindole (Vector Laboratories). All antibodies were diluted in BOND™ primary diluent (Leica). All IHC and IF staining was performed on the Leica BOND RX™ auto-stainer (Leica, Nussloch, Germany).

Human tissues used for positive controls were seminoma for OCT4 and NANOG, skin for SOX2, breast carcinoma for KLF4, and colon for c-MYC. To determine the specificity of the amplification cascade used in IHC staining, negative controls were performed on sections of mHNcSCC tissue samples using a matched isotype control for both mouse (ready-to-use; cat#DK2488, Dako, Glostrup, Denmark) and rabbit (ready-to-use; cat#DK1594, Dako) primary antibodies, and a combination of both was used for determining specificity of the amplification cascade used in IF staining.

### *In-situ* Hybridization

Four micro meter-thick FFPE sections of six mHNcSCC tissue samples from the original cohort of 15 patients, underwent ISH on the Leica BOND RX™ auto-stainer with probes for OCT4 (NM_002701.4) and SOX2 (NR_075091.1), NANOG (NM_024865.2), KLF4 (NM_001314052), and c-MYC (NM_002467.4). All probes used for ISH were obtained from Advanced Cell Diagnostics (Newark, CA, USA). Probes were detected using the RNAscope 2.5 LS Reagent Brown Kit (cat#322100, Advanced Cell Diagnostics).

Human tissues used for positive controls were seminoma for OCT4 and NANOG, skin for SOX2, breast carcinoma for KLF4, and colon for c-MYC. Negative controls were demonstrated on sections of mHNcSCC tissue samples using a probe for DapB (EF191515) (cat#312038, Advanced Cell Diagnostics).

### Image Analysis and Quantification of IHC and ISH Staining

IHC-stained slides were visualized and imaged using an Olympus BX53 light microscope fitted with an Olympus SC100 digital camera (Olympus, Tokyo, Japan), and processed with the cellSens 2.0 Software (Olympus). IF-stained slides were viewed and imaged with an Olympus FV1200 biological confocal laser-scanning microscope and subjected to 2D deconvolutional processing with cellSens Dimension 1.11 software (Olympus).

Cell counting was performed on IHC-stained and ISH-stained slides of mHNcSCC tissue samples using Cell Counter on ImageJ software (National Institutes of Health, Bethesda, MD, USA). Cell counting of IHC-stained slides was performed on three fields of view at 400x magnification, with each field including both the tumor nests (TNs) and the peri-tumoral stroma (PTS) at ~50% of each image. A cell was considered positive for staining if it resembled the positive control for that marker, and was deemed negative for staining if it did not. A cell was deemed positively stained for OCT4, SOX2, NANOG, KLF4, and c-MYC if staining was present in either the nucleus or cytoplasm, and cells were distinguished from one another by the presence of their nuclei and counted. All positively stained cells in the TNs and the PTS for each field were counted and the proportions of positively stained cells out of the total number of cells within the field of view were then calculated and averaged across the three fields of view that had a minimum of 100 cells per field, for each of the 15 cases. Cell counting on ISH-stained slides was performed in the same manner, except the images were taken at 1000x magnification with each view having a minimum of 10 cells, for each of the six cases.

### Reverse-Transcription Quantitative Polymerase Chain Reaction

Total RNA was isolated from four snap-frozen mHNcSCC tissue samples (20 mg/sample) and three mHNcSCC-derived primary cell lines (5 × 10^5^ viable cells/sample) from the original cohort of 15 patients. Tissues were homogenized using the Omni Tissue Homogenizer (Omni TH, Omni International, Kennesaw, GA, USA) before preparation using the RNeasy Mini Kit (cat#74104, Qiagen). Frozen cell pellets were prepared using the RNeasy Micro Kit (cat#74004, Qiagen). A DNase digest step was included for both methods (cat#79254, Qiagen). Quantitation of the RNA was determined using a NanoDrop 2000 Spectrophotometer (Thermo Fisher Scientific). Transcript expression was determined using the Rotor-Gene Q (Qiagen) and the Rotor-Gene Multiplex RT-qPCR Kit (cat#204974, Qiagen). The TaqMan primer probes used were OCT4 (Hs03005111_g1), SOX2 (Hs01053049_s1), NANOG (Hs02387400_g1), KLF4 (Hs00358836_m1), and c-MYC (Hs00153408_m1; cat#4331182). The level of gene expression was normalized to that of the housekeepers GAPDH (Hs99999905_m1) and PUM1 (Hs00206469_m1; cat#4331182), all from Thermo Fisher Scientific. Universal human reference RNA (UHR; cat#636690, Clontech Laboratories, Mountain View, CA, USA) – total RNA from a range of normal adult human tissues, was used as a calibrator for the 2^ΔΔ*Ct*^ analysis. NTERA-2 cells were included as a positive control, and a no template control (NTC) included to control for contamination. End-point amplification products were checked for the presence of bands of the correct size by gel electrophoresis on 2% agarose gels (cat#G402002, Thermo Fisher Scientific). Graphs were generated using GraphPad Prism (v8.0.2, San Diego, CA, USA) and results presented as expression fold change relative to UHR.

### Western Blotting

Total protein extracts from three mHNcSCC-derived primary cell lines were resolved by one-dimensional polyacrylamide gel electrophoresis (Bio-Rad, Hercules, CA, USA) on 4–12% Bis-Tris gels using 20 μg total protein/sample and transferred to polyvinylidene difluoride membranes (Bio-Rad). The membranes were blocked in 1X iBind™ Flex FD Solution (Thermo Fisher Scientific), and then probed with the primary antibodies for c-MYC (1:1,000; cat#Ab32072, Abcam), KLF4 (1:1,000; cat#NBP2-24749, Novus Biologicals), NANOG (1:1,000; cat#Ab109250, Abcam), OCT4 (1:500; cat#Ab109183, Abcam), SOX2 (1:500; cat#48-1400, Thermo Fisher Scientific), and α-tubulin (1:1,000; cat#62204, Thermo Fisher Scientific). They were then incubated with the appropriate secondary antibody, goat anti-rabbit horseradish peroxidase (HRP) conjugate (1:1,000; cat#ab6721, Thermo Fisher Scientific), for the five iPSC markers and goat anti-mouse—Alexa 488 (1:1,000; cat#A21202, Thermo Fisher Scientific) for the loading control (α-tubulin). HRP-conjugated secondary antibody detection was achieved using Clarity Western enhanced chemiluminescence substrate (Bio-Rad) and a ChemiDoc MP imaging system (Bio-Rad).

### *In vitro* Tumorsphere Formation Assays

Tumorsphere suspension cultures were developed using three mHNcSCC-derived primary cell lines. Briefly, 2.5 × 10^5^ live cells from adherent cultures at passages 7–9 were seeded in 24 mL StemXVivo serum-free tumorsphere media (cat#CCM012, R&D Systems, Minneapolis, MN, USA), in T75 Nunclon™ Sphera™ EasYFlasks (cat#174952, Thermo Fisher Scientific) supplemented as per the manufacturer's protocol. Cells were maintained in a 5% incubator at 37°C for up to 10 days, and fed every 3–4 days with the addition of 12 mL of media. Cultures were observed, photographed and measured daily from day 3 post seeding under an Olympus CKX53 Microscope (Tokyo, Japan). Three fields of view were captured for each cell line. From each field of view five representative spheres were identified and two measurements per sphere were performed to establish an average size within each. Culture results are reported as the average size of all spheres measured and the percentage of spheres measured that exceed the 50 μm threshold, which is the minimum required size for a sphere to be considered positive (45).

### Statistical Analysis

Because cell counting was performed for each mHNcSCC tissue sample that underwent IHC staining and ISH staining was tested for all five iPSC markers, and because three technical replicates were done for each marker, a repeated measures analysis was performed to test for differences between markers using Generalized Estimating Equations (GEE) (46). The marginal means and their standard errors were estimated from the GEE model and compared between markers using sequential Bonferroni correction for multiple comparisons. A *p*-value of <0.05 was taken as significant.

## Results

### IHC Staining Showed Expression of iPSC Markers OCT4, NANOG, SOX2, KLF4, and c-MYC in mHNcSCC Tissue Samples

**Figure 1:**
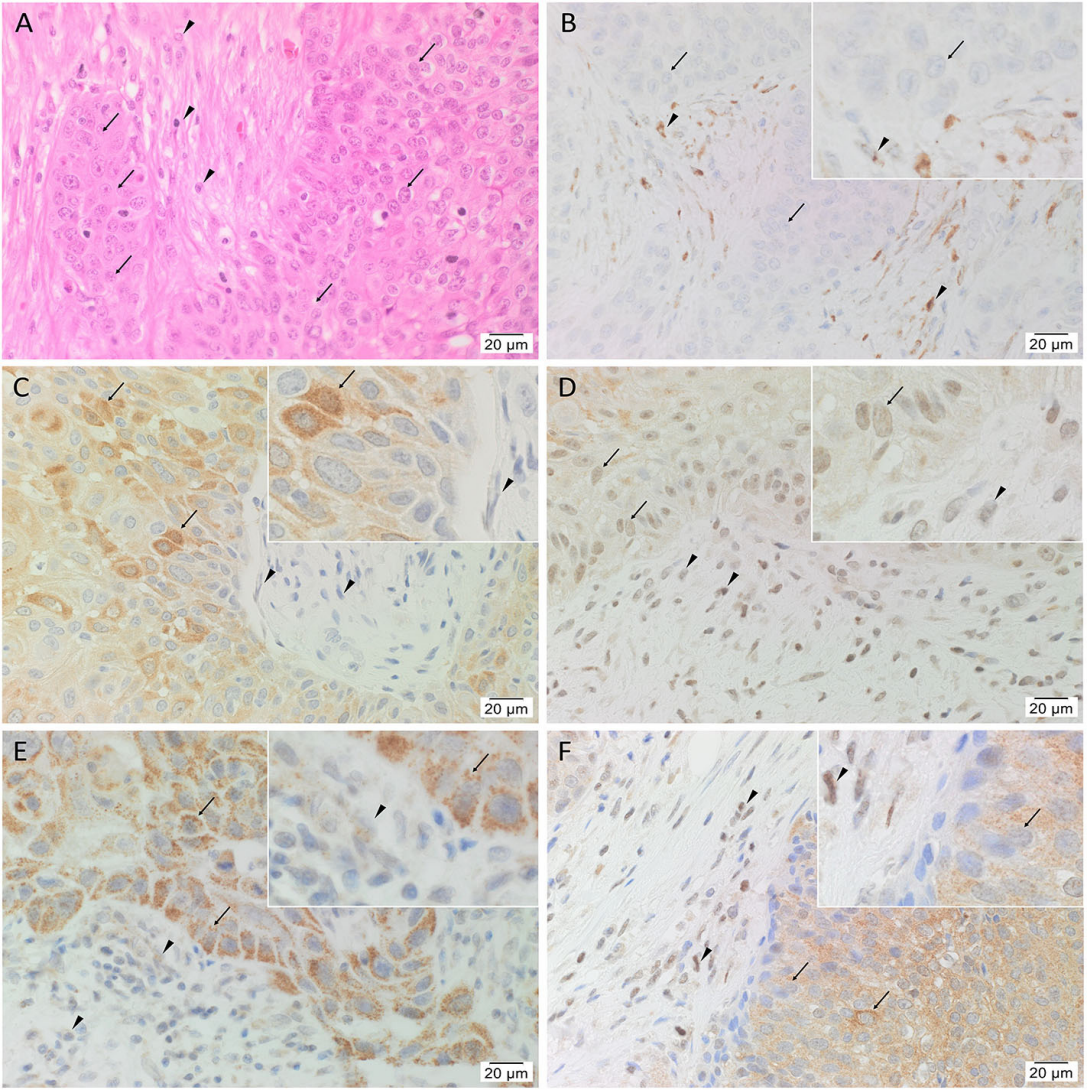
Representative hematoxylin and eosin **(A)** and immunohistochemical **(B–F)** stained slides of metastatic head and neck cutaneous squamous cell carcinoma (mHNcSCC) tissue samples, demonstrating the tumor nests (TNs, *arrows*) separated by peritumoral stroma (PTS, *arrowheads*) **(A)**, and nuclear and cytoplasmic expression of OCT4 (**B**, brown) by cells within the PTS (*arrowheads*), and cytoplasmic expression in occasional cells within the TNs (*arrows*). NANOG (**C**, brown) was expressed on the cytoplasm of cells within the TNs (*arrows*). Cells within the PTS did not express for NANOG (*arrowheads*). SOX2 (**D**, brown) was expressed on the nucleus and the cytoplasm of cells within the TNs (*arrows*) and the PTS (*arrowheads*). KLF4 (**E**, brown) was present on the cytoplasm in cells throughout the TNs (*arrow*), and to a lesser extent, on cells within the PTS (*arrowheads*). c-MYC **(F**, brown) showed cytoplasmic and occasional nuclear expression on cells within the TNs (*arrows*) and cytoplasmic and nuclear expression in cells within the PTS (*arrowheads*). Nuclei were counter-stained with hematoxylin. Original magnification: 400x.

The diagnosis of mHNcSCC was confirmed in all 15 tissue samples showing TNs (*arrows*) surrounded by the PTS (*arrowheads*) (Figure 1A). All 15 mHNcSCC tissue samples exhibited nuclear expression of OCT4 (Figure 1B) by cells within the PTS and faint cytoplasmic staining in occasional cells within the TNs. NANOG (Figure 1C) was expressed in the cytoplasm of cells within the TNs, but not the cells within the PTS. SOX2 (Figure 1D) was expressed on the nucleus and cytoplasm of cells within the TNs, and in the nucleus of cells within the PTS. KLF4 was present in the cytoplasm of cells throughout the TNs (Figure 1E), and to a lesser extent, cells within the PTS (Figure 1E). Cytoplasmic and nuclear expression of c-MYC (Figure 1F) was observed in cells within the TNs and occasional cells within the PTS. Figure insets have been provided to show enlarged views of the corresponding images.

Human tissues for positive controls showed the expected staining patterns: for OCT4 (Supplementary Figure 1A) and NANOG (Supplementary Figure 1B) on seminoma, SOX2 (Supplementary Figure 1C) on skin, KLF4 (Supplementary Figure 1D) on breast carcinoma, and c-MYC (Supplementary Figure 1E) on colon. Specificity of the secondary antibodies was confirmed on sections of mHNcSCC tissue samples using a matched isotype control for both mouse and rabbit primary antibodies (Supplementary Figure 1F).

**Figure 2:**
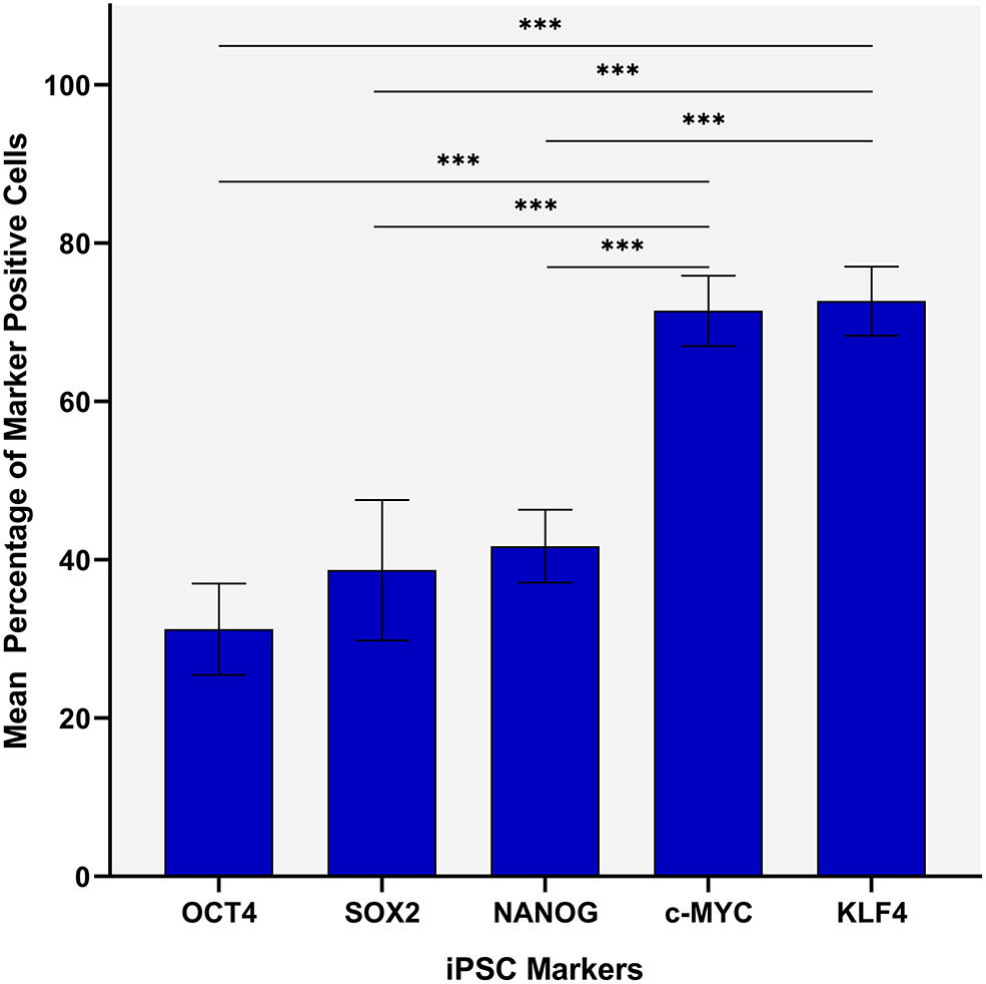
Statistical analyses of cell counting results for immunohistochemical-stained slides of metastatic head and neck cutaneous squamous cell carcinoma demonstrating a statistically significant difference between the mean percentage of cells stained positively in both the TN and the PTS for each induced pluripotent stem cell (iPSC) marker (*p* < 0.0005). Error bars: 95% confidence interval. ***p < 0.05.

Statistical analyses of cell counting results for IHC-stained slides demonstrated a statistically significant difference between the mean proportion of cells that stained positively for each iPSC marker in both the TNs and the PTS (*p* < 0.0005) (Figure 2). The lowest means were found for OCT4 (0.32, 95%CI 0.25–0.39), SOX2 (0.36, 95%CI 0.25–0.48) and NANOG (0.42, 95%CI 0.38–0.47) with no statistically significant difference between them. c-MYC (0.70, 95%CI 0.65–0.75) and KLF4 (0.72, 95%CI 0.67–0.77) had the highest mean proportions of positive cells which were significantly higher than the three aforementioned iPSC markers (Figure 2). There was no statistically significant difference between c-MYC and KLF4 (Figure 2).

### IF Staining Showed Three CSC Subpopulations Within mHNcSCC Tissue Samples

**Figure 3:**
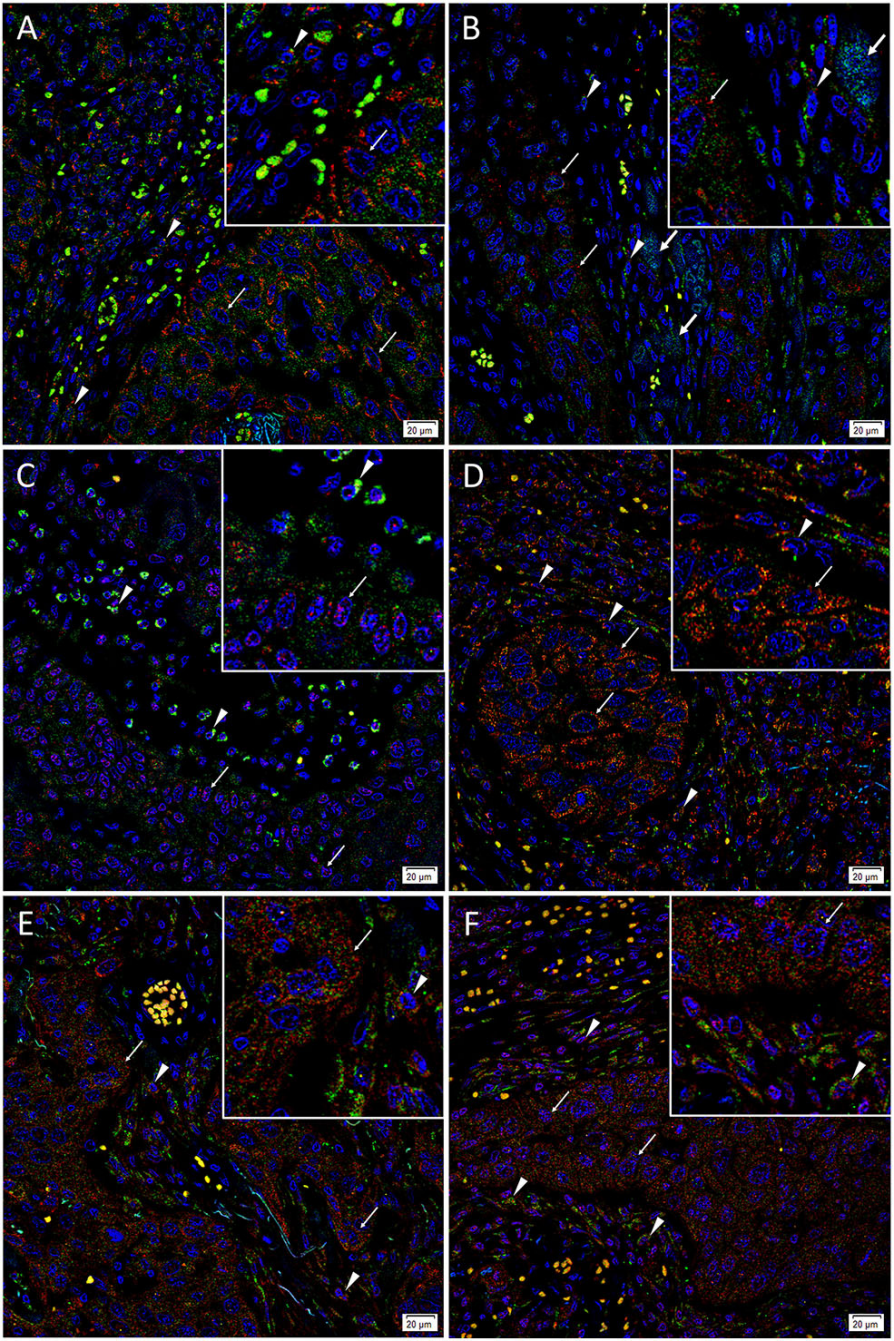
Representative immunofluorescence-stained sections of a metastatic cutaneous head and neck squamous cell carcinoma tissue sample demonstrating expression of induced pluripotent stem cell markers, with abundant expression of c-MYC (**A–C**, green) on the cells within the peritumoral stroma (PTS, *arrowheads*) and the tumor nests (TNs, *thin arrows*). These c-MYC^+^ cells also demonstrated cytoplasmic expression of KLF4 (**A**, red). Most of the c-MYC^+^ cells within the TNs (*thin arrows*) and few of those within the PTS (*arrowheads*) also expressed NANOG (**B**, red) with few c-MYC+ cells within the TNs not expressing NANOG (**B**, red, *thick arrows*). These c-MYC^+^ cells within the TNs (*thin arrows*) and the PTS (*arrowheads*) exhibited nuclear and cytoplasmic expression of SOX2 (**C**, red). The KLF4+ (**D**, red) cells within the TNs (*thin arrows*) and the PTS (*arrowheads*) also expressed OCT4 (**D**, green). The majority of OCT4+ (**E,F**, green) cells within the TNs (*thin arrows*) and the PTS (*arrowheads*) also expressed NANOG (**E**, red), and all expressed SOX2 (**F**, red). All slides were counter-stained with 4′,6′-diamidino-2-phenylindole (**A–F**, blue). Original magnification 400x. The insets show enlarged views of the corresponding images.

IF staining demonstrated abundant expression of c-MYC (Figures 3A–C, green) on the cells within the PTS (*arrowheads*) and the TNs (*arrows*). The c-MYC+ subpopulations within the TNs and the PTS also demonstrated cytoplasmic expression of KLF4 (Figure 3A, red). Most of the c-MYC+ cells within the TNs and few c-MYC+ within the PTS also expressed NANOG (Figure 3B, red) with few c-MYC+ cells within the PTS not expressing NANOG (Figure 3B). These c-MYC+ subpopulations within the TNs and the PTS exhibited nuclear and cytoplasmic expression of SOX2 (Figure 3C, red). The KLF4+ (Figure 3D, red) cells within the TNs and the PTS also expressed OCT4 (Figure 3D, green). The majority of these OCT4+ (Figures 3E,F, green) cells within the TNs and the PTS also expressed NANOG (Figure 3E, red), and all of them expressed SOX2 (Figure 3F, red). Figure insets have been provided to show enlarged views of the corresponding images.

Taken altogether, there is an OCT4+/NANOG +/SOX2+/KLF4+/c-MYC+ CSC subpopulation and an OCT4+/NANOG-/SOX2+/KLF4+/c-MYC+ subpopulation within the TNs, and an OCT4+/NANOG+/SOX2+/KLF4+/c-MYC+ subpopulation within the PTS of mHNcSCC.

Images of individual stains of the merged images presented in Figure 3 are provided in Supplementary Figure 2. Specificity of secondary antibodies was confirmed on the negative control (Supplementary Figure 2M), which demonstrated minimal staining.

### ISH Demonstrated mRNA Transcripts of iPSC Markers in mHNcSCC Tissue Samples

**Figure 4:**
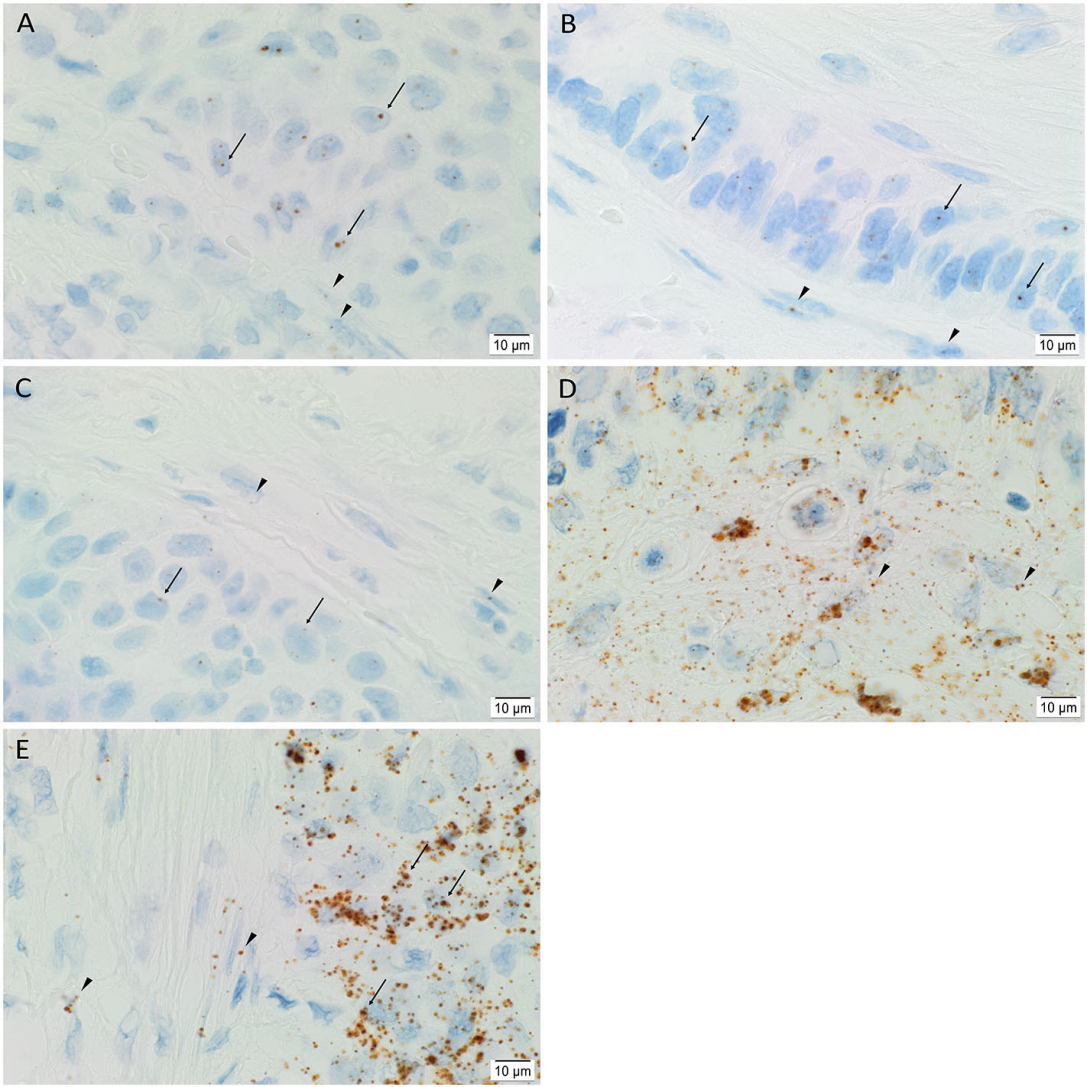
Representative *in situ* hybridization images of a metastatic head and neck cutaneous squamous cell carcinoma tissue sample demonstrating expression of mRNA transcripts of induced pluripotent stem cell markers. Transcripts for OCT4 (**A**, brown), NANOG (**B**, brown), SOX2 (**C**, brown), KLF4 (**D**, brown) and c-MYC (**E**, brown) were present throughout the TNs (*arrows*), and to a lesser degree, in the PTS (*arrowheads*). Nuclei were counter-stained with hematoxylin (**A–E**, blue). Original magnification: 1000x.

ISH showed mRNA expression of OCT4 (Figure 4A), NANOG (Figure 4B), SOX2 (Figure 4C), KLF4 (Figure 4D), and c-MYC (Figure 4E) throughout the TNs (*arrows*), and to a lesser extent, the PTS (*arrowheads*), in all six mHNcSCC samples examined.

Human tissues used for positive controls demonstrated positive staining of OCT4 (Supplementary Figure 3A) and NANOG (Supplementary Figure 3B) on sections of seminoma, SOX2 (Supplementary Figure 3C) on skin, KLF4 (Supplementary Figure 3D) on breast carcinoma, and c-MYC (Supplementary Figure 3E) on colon. The negative control (Supplementary Figure 3F) on sections of mHNcSCC tissue samples confirmed the specificity of the primary antibodies.

**Figure 5:**
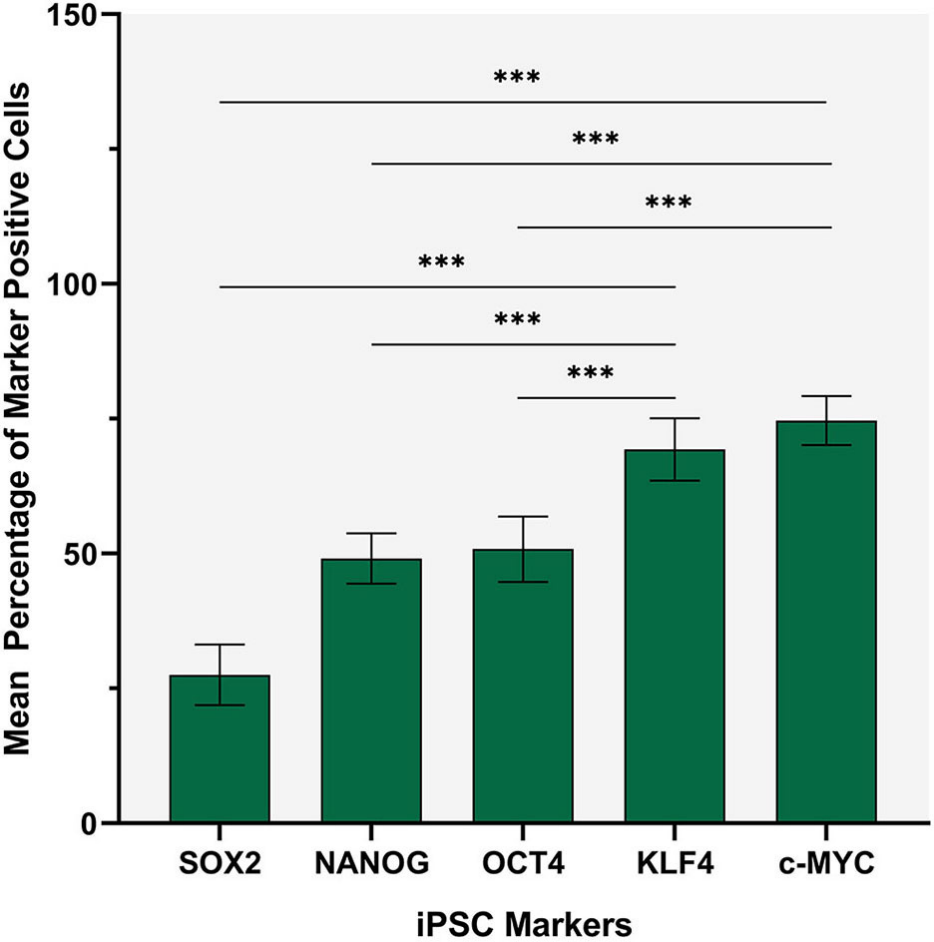
Statistical analysis of cell counting for *in situ* hybridization stained slides demonstrating that c-MYC had the highest mean proportion of positive cells, followed by KLF4 although there was no statistically significant difference between them. OCT4 and NANOG had lower mean proportions of positive cells compared with c-MYC and KLF4 although there was no statistically significance difference between them. The mean proportion of cells that stained positively for SOX2 was significantly lower (*p* < 0.0005) than the other four iPSC markers. Error bars: 95% confidence interval. ***p < 0.05.

Statistical analyses of the cell counting results for the ISH-stained slides demonstrated a statistically significant difference between the mean proportion of cells that stained positively for each iPSC marker (*p* < 0.0005) (Figure 5). c-MYC (0.70, 95%CI 0.67–0.73) had highest mean proportion of positive cells, followed by KLF4 (0.68, 95%CI 0.66–0.71) although there was no statistically significant difference between them. OCT4 (0.54, 95%CI 0.49–0.59) and NANOG (0.48, 95%CI 0.47–0.50) had significantly lower mean proportions of positive cells compared with c-MYC and KLF4, but were not significantly different from each other (*p* < 0.0005) (Figure 5). The mean proportion of cells that stained positively for SOX2 was significantly lower than the other four iPSC markers (0.31, 95%CI 0.26–0.36) (*p* < 0.0005) (Figure 5).

### RT-qPCR Demonstrated mRNA Transcripts of iPSC Markers in mHNcSCC Tissue Samples and mHNcSCC-Derived Primary Cell Lines

**Figure 6:**
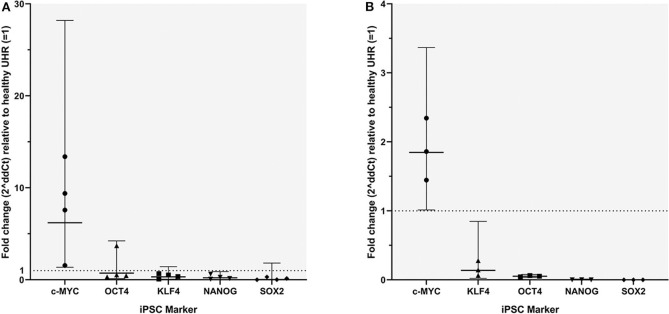
Graph showing 2^ΔΔCT^ values of RT-qPCR runs performed on four snap-frozen metastatic head and neck cutaneous squamous cell carcinoma (mHNcSCC) tissue samples **(A)** and four mHNcSCC-derived primary cell lines **(B)**, amplifying transcripts for OCT4, NANOG, SOX2, KLF4, and c-MYC. ΔΔCT was calculated by normalizing CT values of iPSC markers to that of the housekeeping genes GAPDH and PUM1, and then expressing this relative to the ΔCT of normal UHR. Error bars: 95% confidence interval.

RT-qPCR analysis demonstrated transcript expression of OCT4, NANOG, SOX2, KLF4, and c-MYC in all four mHNcSCC tissue samples (Figure 6A) and in all three of the mHNcSCC-derived primary cell lines (Figure 6B).

In the mHNcSCC tissue samples c-MYC showed a biologically significant increase in expression, relative to UHR. SOX2 had a biologically significant (increase in expression, relative to UHR. SOX2 had a biologically significant decrease in expression, relative to UHR. The markers with the highest to the lowest mRNA expression were: c-MYC > OCT4, KLF4 ≥ NANOG ≥ SOX2. In the mHNcSCC-derived primary cell lines, OCT4, NANOG, and SOX2 all showed a biologically relevant decrease in expression, relative to UHR. The markers with the highest to the lowest mRNA expression were: c-MYC > KLF4 > OCT4 > NANOG ≥ SOX2.

Specific amplification of the products was demonstrated by electrophoresis of qPCR products on 2% agarose gels (Supplementary Figure 4). The expected size amplicons were observed, and no products were observed in the NTC reactions (Supplementary Figure 4).

### Western Blotting Demonstrated Expression of SOX2, KLF4, and c-MYC but Not OCT4 and NANOG by mHNcSCC-Derived Primary Cell Lines

**Figure 7:**
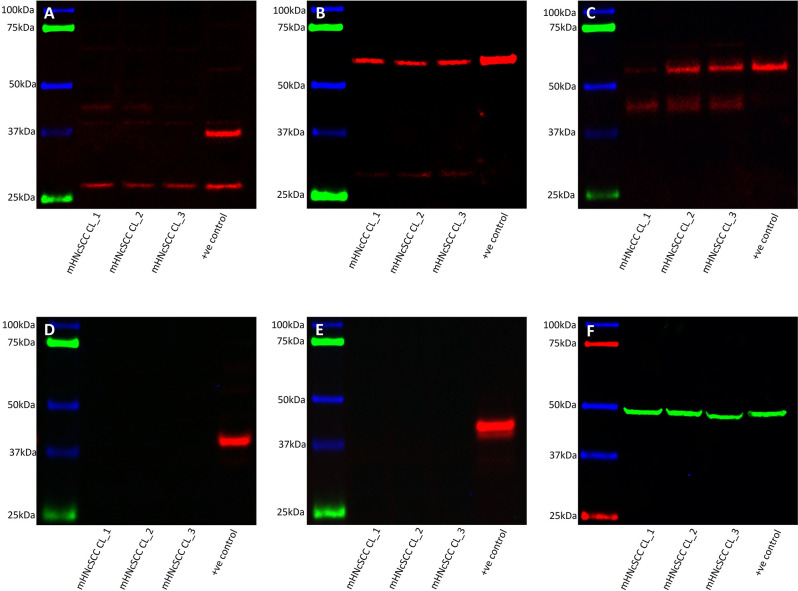
Western blot analysis of three metastatic cutaneous head and neck squamous cell carcinoma-derived primary cell lines. This demonstrated the presence of SOX2 **(A)**, KLF4 **(B)**, and c-MYC **(C)** in all three cell lines. NANOG **(D)**, and OCT4 **(E)** were not detected. Approximately equal amounts of protein were loaded into each lane, as confirmed by α-tubulin **(F)**.

WB of mHNcSCC-derived primary cell lines derived from mHNcSCC tissue samples from three of the original cohort of 15 patients showed very low expression levels of SOX2 over two bands: one at the expected 35 kDa size and another at 40–42 kDa (Figure 7A), which is consistent with one of its known post-translational modifications (47). KLF4 (Figure 7B) and c-MYC (seen as two bands) (Figure 7C) were expressed in all three mHNcSCC-derived primary cell lines; the lower band for c-MYC may be MYC-nick (48). NANOG (Figure 7D) and OCT4 (Figure 7E) were below detectable levels. Equal amounts of proteins were loaded into each lane, as confirmed by α-tubulin staining (Figure 7F).

### mHNcSCC-Derived Primary Cell Lines Demonstrated Tumorsphere Formation *in vitro*

**Figure 8:**
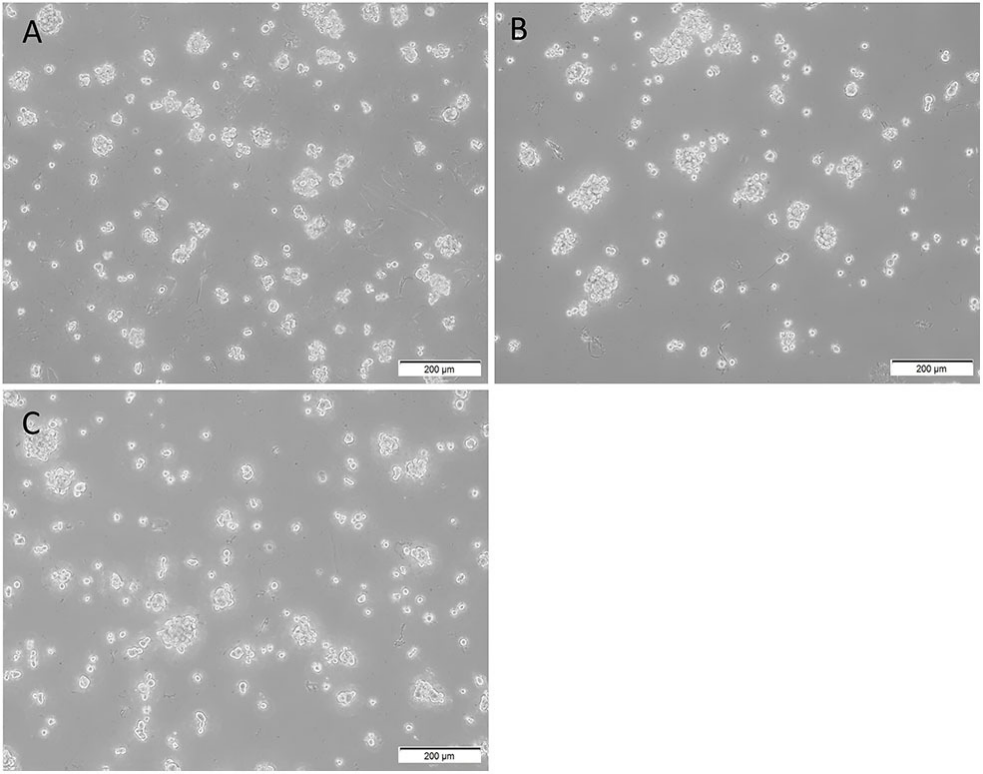
Representative low magnification (10x) images of tumorsphere formation of the three metastatic head and neck cutaneous squamous cell carcinoma (mHNcSCC)-derived primary cell lines, averaging 62.6, 72.9, and 88.7 μm in diameter at the onset of dark centers, respectively **(A–C)**.

All three mHNcSCC-derived primary cell lines demonstrated *in vitro* tumorsphere formation (Figures 8A–C) at the first passage with a mean diameter at cessation of culture averaging 62.6, 72.9, and 88.7 μm respectively (Supplementary Figure 5), meeting our threshold (spheres that were > 50 μm in size). The percentage of spheres measured that exceeded the 50 μm threshold in these cell lines were 93.3, 100, and 100%, respectively (45). Tumorsphere culture was halted at the first signs of the on-set of dark centers indicating cell death. This occurred on days 6, 7, and 5 respectively. This provides some preliminary evidence of stem cell functionality within the mHNcSCC-derived primary cell lines.

## Discussion

NMSCs are the commonest cancers diagnosed worldwide (49) and make up one third of all malignancies (50), with 20% being SCC (51).

Tumor initiation (23), development (52), recurrence (53), and chemoradiation resistance have been attributed to the presence of CSCs (54), which have been recently identified within primary HNcSCC (19) using the five iPSC markers used in this study. Targeting CSCs has been suggested as a novel treatment approach for cancer (55).

In this study, we identified three CSC subpopulations within mHNcSCC: an OCT4+/NANOG+/SOX2+/KLF4+/c-MYC+ subpopulation and an OCT4+/NANOG-/SOX2+/KLF4+/c-MYC+ subpopulation within the TNs, and an OCT4+/NANOG+/SOX2+/KLF4+/c-MYC+ subpopulation within the PTS.

We have previously identified an OCT4+/NANOG+ /SOX2+/KLF4+/c-MYC+ CSC subpopulation within the TNs, and an OCT4+/NANOG+/SOX2+/KLF+/c-MYC+ and an OCT4+/NANOG-/SOX2+/KLF4+/c-MYC+ CSC subpopulation within the PTS of primary HNcSCC (19). The CSC subpopulations within primary HNcSCC differ from the CSC subpopulations identified in mHNcSCC demonstrated in this study. We have identified an OCT4+/NANOG-/SOX2+/KLF4+/c-MYC+ population within the TNs in mHNcSCC, which is not present in primary HNcSCC. The OCT4+/NANOG-/SOX2+/KLF4+/c-MYC+ CSC subpopulation identified within the PTS in primary HNcSCC, is not demonstrated in mHNcSCC in this study. Using the same iPSC markers, we have also shown the presence of two CSC subpopulations within head and neck metastatic malignant melanoma (HNmMM): an OCT4+/SOX2+/KLF4+/c-MYC+ CSC subpopulation within the TNs, and another in the PTS with NANOG present only in two of the 20 cases studied (44). Three out of four primary cell lines derived from four different HNmMM tissue samples formed tumorspheres *in vitro*. As with the current study of mHNcSCC, the formation of tumorspheres in HNmMM-derived primary cell lines *in vitro*, provides preliminary functional evidence of the presence of CSCs in HNmMM tissues (44).

The presence of the CSC subpopulation within the PTS of mHNcSCC was supported by both protein and mRNA expression of OCT4, NANOG, SOX2, KLF4, and c-MYC on the cells within the PTS by IHC staining and ISH, respectively. The absence of NANOG in one of the two CSC subpopulations within the TNs aligns with previous studies demonstrating dispensability of NANOG in the presence of the other four iPSC markers for induction of pluripotency (13), the phenotype exhibited by CSCs (8). As with primary HNcSCC (19), IHC staining in this study demonstrated localization of NANOG to CSCs in the TNs, and ISH detected mRNA transcripts for NANOG in cells within the TNs and, to a lesser extent, those within the PTS. The presence of NANOG mRNA in cells within the TNs and the absence of its protein in one of the two CSC subpopulations within the TNs, is consistent with the dispensability of NANOG for pluripotent capability (14). The absence of NANOG in the aforementioned CSC subpopulation is interesting, as NANOG has been associated with the development of cervical SCC when it is present on stromal cells (56). NANOG has been used as a CSC marker in many cancer types (16, 37–40, 57). Targeting NANOG in head and neck squamous cell carcinoma-derived spheroid cells significantly inhibits tumor aggressiveness and increases chemosensitivity to cisplatin (57); perhaps targeting NANOG within mHNcSCC would have a similar effect on these parameters.

The presence of CSCs within mHNcSCC tissue samples is supported by RT-qPCR which demonstrated the presence of all five iPSC markers in three mHNcSCC-derived primary cell lines, while WB showed expression of these markers except for NANOG and OCT4. The capability of the three mHNcSCC-derived primary cell lines to form initial tumorspheres *in vitro* provides preliminary evidence of the presence of CSCs within mHNcSCC. Further functional tests to confirm these findings, such as additional passages and *in vivo* xenotransplantation of the said tumorspheres into mice, should be undertaken.

As with our previous study of primary HNcSCC (19), SOX2 expression was observed within mHNcSCC tissue samples and mHNcSCC-derived primary cell lines, albeit at very low levels. This may suggest the dispensability of SOX2 following metastasis, as observed in metastatic malignant melanoma (58).

If SOX2 is dispensable following metastasis in mHNcSCC as in metastatic malignant melanoma (58), a correlation between low SOX2 expression levels and a more aggressive cancer may be expected. However, Li et al. (59) demonstrate that SOX2 is a downstream target of the Hippo effector TAZ in HNcSCC, which is capable of reprogramming differentiated cancer cells into CSCs, and that SOX2 and TAZ upregulation are both associated with poorer overall survival in HNcSCC. Given the presence of SOX2 in mHNcSCC (60) and its association with local recurrence and metastasis, we speculate the TAZ-SOX2 axis as an important determinant of CSC stemness in this tumor. The low expression of SOX2 within mHNcSCC-derived primary cell lines and the lower proportions of SOX2 positive cells in mHNcSCC tissue samples on IHC staining and ISH staining compared to the other four iPSC markers may reflect the aggressiveness of this cancer, as decreased SOX2 expression has been associated with carcinogenesis and the development of OCSCC (60).

RT-qPCR and WB analyses showed some differences of the expression profile in the iPSC markers between the mHNcSCC tissue samples and mHNcSCC-derived primary cell lines. It has been observed that the expression of up to 10% of genes is altered within five passages during tumorsphere formation *in vitro* (61). These alterations in gene expression driven by the cell culture environment may explain why SOX2 was expressed at a very low level, and why OCT4 and NANOG were not detected by WB in mHNcSCC-derived primary cell lines, compared with the RT-qPCR results for the mHNcSCC tissue samples. It may also account for the differences in the RT-qPCR results between the mHNcSCC tissue samples and the mHNcSCC-derived primary cell lines.

Given its role and overexpression in cSCC (62), it was unsurprising that KLF4 was expressed in all mHNcSCC tissue samples and mHNcSCC-derived primary cell lines. Our finding of c-MYC expression in mHNcSCC aligns with reports of c-MYC overexpression in other types of carcinoma (29, 37, 39, 40).

The finding of OCT4 expression in mHNcSCC is consistent with our previous study demonstrating OCT4 expression in primary HNcSCC (19) and other solid tumors (63). As a key regulator of pluripotency in the early mammalian embryo and given it is consistently expressed by ESCs and hematopoietic and neural stem cells (26), the presence of OCT4 in mHNcSCC supports the presence of primitive CSC subpopulations in this tumor.

Expression of components of the renin-angiotensin system (RAS) by CSCs has been demonstrated in different cancer types including glioblastoma (64), OCSCC affecting the oral tongue (65), buccal mucosa (66), and lip (67) squamous cell carcinoma (SCC), primary HNcSCC (68), metastatic colon adenocarcinoma (42), and metastatic malignant melanoma (43). This suggests CSCs in these tumors may be targeted by modulation of the RAS (12, 55).

We have demonstrated the presence of three putative CSC subpopulations within mHNcSCC. Further, investigating the expression of the RAS—an important regulator of stem cells (69, 70)—by these CSC subpopulations may provide an avenue of therapeutic targeting of them in the treatment of this aggressive cancer (55). These findings are novel, and further investigation with a larger sample size with functional studies are needed to confirm the CSC subpopulations identified in this study.

## Conclusions

This study demonstrates expression of the iPSC markers OCT4, NANOG, SOX2, KLF4, and c-MYC in mHNcSCC, with the presence of three CSC subpopulations. mHNcSCC-derived primary cell lines express transcripts of OCT4, NANOG, SOX2, KLF4 and c-MYC. SOX2, KLF4, and c-MYC, but not OCT4 and NANOG proteins. These cell lines form tumorspheres *in vitro*, at a single passage. The identification of CSCs within mHNcSCC may open novel avenues for therapeutic targeting of this aggressive cancer.

## Data Availability Statement

The datasets generated for this study are available on request to the corresponding author.

## Ethics Statement

The studies involving human participants were reviewed and approved by Central Regional Health and Disability Ethics Committee (Ref. 12/CEN/74AM05). The patients provided their written informed consent to participate in this study.

## Author Contributions

TI and ST formulated the study hypothesis and design. EK, HB, PD, TI, and ST interpreted the immunohistochemical data. EK, NB, TI, and ST interpreted the immunofluorescence data. NB performed the WB analysis. NB, EK, and ST interpreted the WB data. BS and JP performed the RT-qPCR experiments. BS, JP, EK, and ST interpreted the RT-qPCR data. EP performed cell culture and tumorsphere experiments and interpreted the data. DS carried out statistical analysis. EK and ST drafted the manuscript. PD, IR, and ST critically revised the manuscript. All authors contributed to the article and approved the submitted version.

## Conflict of Interest

TI, PD, and ST are inventors of the patents Cancer Diagnosis and Therapy (PCT/NZ2015/050108), Cancer Therapeutic (PCT/NZ2018/050006), Novel Pharmaceutical Compositions for Cancer Therapy (US/62/711709), and Cancer diagnosis and therapy (United States Patent No. 10281472). The authors of this manuscript have no external sources of funding to disclose. The remaining authors declare that the research was conducted in the absence of any commercial or financial relationships that could be construed as a potential conflict of interest.

## References

[1] Brougham NDL Tan ST. The incidence and risk factors of metastasis for cutaneous squamous cell carcinoma—implications on the T-classification system. J Surg Oncol. (2014) 110:876–82. 10.1002/jso.23731 25088537

[2] Ch'ng S Maitra A Allison R Chaplin J Gregor RT Lea R. Parotid and cervical nodal status predict prognosis for patients with head & neck metastatic cutaneous squamous cell carcinoma - a multicentre study of the New Zealand population. J Surg Oncol. (2008) 98:101–5. 10.1002/jso.21092 18523982

[3] Rudolph R Zelac DE. Squamous cell carcinoma of the skin. Plast Reconstr Surg. (2004) 114:82e−94e. 10.1097/01.PRS.0000138243.45735.8A 15509920

[4] Brougham NDL Dennett ER Cameron R Tan ST. Incidence and risk factors of metastasis for cutaneous squamous cell carcinoma. J Surg Oncol. (2012) 106:811–5. 10.1002/jso.23155 22592943

[5] Brougham NDL Dennett ER Tan ST. Changing incidence of non-melanoma skin cancer in New Zealand. ANZ J Surg. (2011) 81:633–6. 10.1111/j.1445-2197.2010.05583.x 22295395

[6] Ch'ng S Maitra A Lea R Brasch H Tan ST. Parotid metastasis – an independent prognostic factor for head and neck cutaneous squamous cell carcinoma. J Plast Reconstr Aesth Surg. (2006) 59:1288–93. 10.1016/j.bjps.2006.03.043 17113505

[7] Xu R Cai MY Luo RZ Tian X Chen MK. The expression status and prognostic value of cancer stem cell biomarker CD133 in cutaneous squamous cell carcinoma. JAMA Dermatol. (2016) 152:305–11. 10.1001/jamadermatol.2015.3781 26560495

[8] Batlle E Clevers H. Cancer stem cells revisited. Nat Med. (2017) 23:1124–34. 10.1038/nm.4409 28985214

[9] Jordan CT Guzman ML Noble M. Cancer stem cells. N Engl J Med. (2006) 355:1253–61. 10.1056/NEJMra061808 16990388

[10] Clement V Sanchez P De Tribolet N Radovanovic I Altaba AR. HEDGEHOG-GLI1 signaling regulates human glioma growth, cancer stem cell self-renewal, and tumorigenicity. Curr Biol. (2007) 17:165–72. 10.1016/j.cub.2006.11.033 17196391 PMC1855204

[11] Gupta PB Chaffer CL Weinberg RA Cancer stem cells: mirage or reality? Nat Med. (2009) 15:1010–2. 10.1038/nm0909-1010 19734877

[12] Munro MJ Wickremesekera AC Davis PF Marsh R Tan ST Itinteang T Renin-angiotensin system and cancer: a review. Integr Cancer Sci Ther. (2017) 4:1–6. 10.15761/ICST.1000231

[13] Takahashi K Yamanaka S. Induction of pluripotent stem cells from mouse embryonic and adult fibroblast cultures by defined factors. Cell. (2006) 126:663–76. 10.1016/j.cell.2006.07.024 16904174

[14] Takahashi K Tanabe K Ohnuki M Narita M Ichisaka T Tomoda K. Induction of pluripotent stem cells from adult human fibroblasts by defined factors. Cell. (2007) 131:861–72. 10.1016/j.cell.2007.11.019 18035408

[15] Yu J Vodyanik MA Smuga-Otto K Antosiewicz-Bourget J Frane JL Tian S. Induced pluripotent stem cell lines derived from human somatic cells. Science. (2007) 318:1917–20. 10.1126/science.1151526 18029452

[16] Humphries HN Wickremesekera SK Marsh RW Brasch HD Mehrotra S Tan ST. Characterization of cancer stem cells in colon adenocarcinoma metastasis to the liver. Front Surg. (2018) 22:76. 10.3389/fsurg.2017.00076 29404335 PMC5786574

[17] Cane R Kennedy-Smith A Brasch HD Savage S Marsh RW Tan ST Characterization of cancer stem cells in renal clear cell carcinoma. Stem Cell Regen Biol. (2019) 4:6–16. 10.15436/2471-0598.19.2462

[18] Munro MJ Wickremesekera SK Peng L Marsh RW Itinteang T Tan ST. Cancer stem cell subpopulations in primary colon adenocarcinoma. PLoS ONE. (2019) 14:e0221963. 10.1371/journal.pone.0221963 31491003 PMC6730900

[19] Koh SP Brasch HD de Jongh J Itinteang T Tan ST. Cancer stem cell subpopulations in moderately differentiated head and neck cutaneous squamous cell carcinoma. Heliyon. (2019) 5:e02257. 10.1016/j.heliyon.2019.e02257 31463389 PMC6709152

[20] Avilion AA Nicolis SK Pevny LH Perez L Vivian N Lovell-Badge R. Multipotent cell lineages in early mouse development depend on SOX2 function. Genes Dev. (2003) 17:126–40. 10.1101/gad.224503 12514105 PMC195970

[21] Siegle JM Basin A Sastre-Perona A Yonekubo Y Brown J Sennett R. SOX2 is a cancer-specific regulator of tumour initiating potential in cutaneous squamous cell carcinoma. Nat Commun. (2014) 5:4511. 10.1038/ncomms5511 25077433 PMC4207965

[22] Lien WH Guo X Polak L Lawton LN Young RA Zheng D. Genome-wide maps of histone modifications unwind *in vivo* chromatin states of the hair follicle lineage. Cell Stem Cell. (2011) 9:219–32. 10.1016/j.stem.2011.07.015 21885018 PMC3166618

[23] Boumahdi S Driessens G Lapouge G Rorive S Nassar D Le Mercier M. SOX2 controls tumour initiation and cancer stem-cell functions in squamous-cell carcinoma. Nature. (2014) 511:246–50. 10.1038/nature13305 24909994

[24] Cauffman G Van de Velde H Liebaers I Van Steirteghem A. Oct-4 mRNA and protein expression during human preimplantation development. Mol Hum Reprod. (2004) 11:173–81. 10.1093/molehr/gah155 15695770

[25] Ding J Xu H Faiola F Ma'ayan A Wang J. Oct4 links multiple epigenetic pathways to the pluripotency network. Cell Res. (2012) 22:155–67. 10.1038/cr.2011.179 22083510 PMC3252465

[26] Koo BS Lee SH Kim JM Huang S Kim SH Rho YS. Oct4 is a critical regulator of stemness in head and neck squamous carcinoma cells. Oncogene. (2015) 34:2317–24. 10.1038/onc.2014.174 24954502

[27] Chiou SH Yu CC Huang CY Lin SC Liu CJ Tsai TH. Positive correlations of Oct-4 and Nanog in oral cancer stem-like cells and high-grade oral squamous cell carcinoma. Clin Cancer Res. (2008) 14:4085–95. 10.1158/1078-0432.CCR-07-4404 18593985

[28] Chambers I Silva J Colby D Nichols J Nijmeijer B Robertson M. Nanog safeguards pluripotency and mediates germline development. Nature. (2007) 450:1230–4. 10.1038/nature06403 18097409

[29] Munro MJ Wickremesekera SK Peng L Tan ST Itinteang T. Cancer stem cells in colorectal cancer: a review. J Clin Pathol. (2018) 71:110–6. 10.1136/jclinpath-2017-204739 28942428

[30] Bourillot PY Savatier P. Krüppel-like transcription factors and control of pluripotency. BMC Biol. (2010) 8:125. 10.1186/1741-7007-8-125 20875146 PMC2946285

[31] Laurenti E Wilson A Trumpp A. Myc's other life: stem cells and beyond. Curr Opin Cell Biol. (2009) 21:844–54. 10.1016/j.ceb.2009.09.006 19836223

[32] Dang CV. MYC, metabolism, cell growth, and tumorigenesis. Cold Spring Harb Perspect Med. (2013) 3:a014217. 10.1101/cshperspect.a014217 23906881 PMC3721271

[33] Royds JA Sharrard RM Wagner B Polacarz SV. Cellular localisation of c-MYC product in human colorectal epithelial neoplasia. J Pathol. (1992) 166:225–33. 10.1002/path.1711660304 1381423

[34] Royds JA Sharrard RM Parsons MA Lawry J Rees R Cottam D. c-MYC oncogene expression in ocular melanomas. Graefe's Arch Clin Exp Opthamol. (1992) 230:366–71. 10.1007/BF00165947 1505770

[35] Lee KM Giltnane JM Balko JM Schwarz LJ Guerrero-Zotano AL Hutchinson KE. MYC and MCL1 cooperatively promote chemotherapy-resistant breast cancer stem cells via regulation of mitochondrial oxidative phosphorylation. Cell Metab. (2017) 26:633–47. 10.1158/1538-7445.AM2016-3328 28978427 PMC5650077

[36] Yin XY Grove L Datta NS Katula K Long MW Prochownik V. Inverse regulation of cyclin B1 by c-Myc and p53 and induction of tetraploidy by cyclin B1 overexpression. Cancer Res. (2001) 61:6487–93. 11522645

[37] van Schaijik B Davis PF Wickremesekera AC Tan ST Itinteang T. Subcellular localisation of the stem cell markers OCT4, SOX2, NANOG, KLF4 and c-MYC in cancer: a review. J Clin Pathol. (2018) 71:88–91. 10.1136/jclinpath-2017-204815 29180509

[38] Yu HH Featherston T Tan ST Chibnall AM Brasch HD Davis PF Characterization of cancer stem cells in moderately differentiated buccal mucosal squamous cell carcinoma. Front Surg. (2016) 3:46 10.3389/fsurg.2016.00046 27532037 PMC4970507

[39] Ram R Brasch HD Dunne JC Davis PF Tan ST Itinteang T. The identification of three cancer stem cell subpopulations within moderately differentiated lip squamous cell carcinoma. Front Surg. (2017) 4:12. 10.3389/fsurg.2017.00012 28321397 PMC5337496

[40] Baillie R Itinteang T Helen HY Brasch HD Davis PF Tan ST. Cancer stem cells in moderately differentiated oral tongue squamous cell carcinoma. J Clin Pathol. (2016) 69:742–4. 10.1136/jclinpath-2015-203599 27095085 PMC4975854

[41] Bradshaw A Wickremesekera A Brasch HD Chibnall AM Davis PF Tan ST Cancer stem cells in glioblastoma multiforme. Front Surg. (2016) 3:48 10.3389/fsurg.2016.00048 27617262 PMC5001191

[42] Narayanan A Wickremesekera SK van Schaijik B Marsh RW Brasch HD Tan ST Cancer stem cells in liver metastasis from colorectal adenocarcinoma express components of the renin-angiotensin system. J Cancer Metastasis Treat. (2019) 5:36 10.20517/2394-4722.2018.77

[43] Wickremesekera AC Brasch HD Lee VM Davis PF Woon K Johnson R. Expression of cancer stem cell markers in metastatic melanoma to the brain. J Clin Neurosci. (2019) 60:112–6. 10.1016/j.jocn.2018.10.068 30626524

[44] Yoganandarajah V Patel J van Schaijik B Bockett N Brasch HD Paterson E. Identification of cancer stem cell subpopulations in head and neck metastatic malignant melanoma. Cells. (2020) 9:e324. 10.3390/cells9020324 32019273 PMC7072148

[45] Lee CH Yu CC Wang BY Chang WW. Tumorsphere as an effective *in vitro* platform for screening anti-cancer stem cell drugs. Oncotarget. (2016) 7:1215–26. 10.18632/oncotarget.6261 26527320 PMC4811455

[46] Hanley JA Negassa A Edwardes MD Forrester JE. Statistical analysis of correlated data using generalized estimating equations: an orientation. Am J Epidemiol. (2003) 157:364–75. 10.1093/aje/kwf215 12578807

[47] Chao AT Jones WM Bejsovec A. The HMG-box transcription factor SoxNeuro acts with Tcf to control Wg/Wnt signaling activity. Development. (2007) 134:989–97. 10.1242/dev.02796 17267442

[48] Conacci-Sorrell M Ngouenet C Eisenman RN. Myc-nick: a cytoplasmic cleavage product of Myc that promotes α-tubulin acetylation and cell differentiation. Cell. (2010) 142:480–93. 10.1016/j.cell.2010.06.037 20691906 PMC2923036

[49] Staples MP Elwood M Burton RC Williams JL Marks R Giles GG. Non-melanoma skin cancer in Australia: the 2002 national survey and trends since 1985. Med J Aust. (2006) 184:6–10. 10.5694/j.1326-5377.2006.tb00086.x 16398622

[50] Surdu S. Non-melanoma skin cancer: occupational risk from UV light and arsenic exposure. Rev Environ Health. (2014) 29:255–65. 10.1515/reveh-2014-0040 25222586

[51] Burton KA Ashack KA Khachemoune A. Cutaneous squamous cell carcinoma: a review of high-risk and metastatic disease. Am J Clin Dermatol. (2016) 17:491–508. 10.1007/s40257-016-0207-3 27358187

[52] Cabrera MC Hollingsworth RE Hurt EM. Cancer stem cell plasticity and tumor hierarchy. World J Stem Cells. (2015) 7:27–36. 10.4252/wjsc.v7.i1.27 25621103 PMC4300934

[53] Peitzsch C Tyutyunnykova A Pantel K Dubrovska A. Cancer stem cells: the root of tumor recurrence and metastases. Semin Cancer Biol. (2017) 44:10–24. 10.1016/j.semcancer.2017.02.011 28257956

[54] Ishii H Iwatsuki M Ieta K Ohta D Haraguchi N Mimori K. Cancer stem cells and chemoradiation resistance. Cancer Sci. (2008) 99:1871–7. 10.1111/j.1349-7006.2008.00914.x 19016744 PMC11159283

[55] Roth I Wickremesekera AC Wickremesekera SK Davis PF Tan ST. Therapeutic targeting of cancer stem cells via modulation of the renin-angiotensin system. Front Oncol. (2019) 9:745. 10.3389/fonc.2019.00745 31440473 PMC6694711

[56] Gu TT Liu SY Zheng PS. Cytoplasmic NANOG-positive stromal cells promote human cervical cancer progression. Am J Pathol. (2012) 181:652–61. 10.1016/j.ajpath.2012.04.008 22683467

[57] Huang CE Yu CC Hu FW Chou MY Tsai LL. Enhanced chemosensitivity by targeting Nanog in head and neck squamous cell carcinomas. Int J Mol Sci. (2014) 15:14935–48. 10.3390/ijms150914935 25158233 PMC4200775

[58] Schaefer S Segalada C Cheng PF Bonalli M Parfejevs V Levesque MP. Sox2 is dispensable for primary melanoma and metastasis formation. Oncogene. (2017) 36:4516–24. 10.1038/onc.2017.55 28368416

[59] Li J Li Z Wu Y Wang Y Wang D Zhang W. The hippo effector TAZ promotes cancer stemness by transcriptional activation of SOX2 in head neck squamous cell carcinoma. Cell Death Dis. (2019) 10:1–15. 10.1038/s41419-019-1838-0 31399556 PMC6689034

[60] Naini FB Shakib PA Abdollahi A Hodjat M Mohammadpour H Khoozestani NK. Relative expression of OCT4, SOX2 and NANOG in oral squamous cell carcinoma versus adjacent non-tumor tissue. Asian Pac J Cancer Prev. (2019) 20:1649–54. 10.31557/APJCP.2019.20.6.1649 31244283 PMC7021633

[61] Neumann E Riepl B Knedla A Lefevre S Tarner IH Grifka J. Cell culture and passaging alters gene expression pattern and proliferation rate in rheumatoid arthritis synovial fibroblasts. Arthritis Res Ther. (2010) 12:R83. 10.1186/ar3010 20462438 PMC2911867

[62] Foster KW Liu Z Nail CD Li X Fitzgerald TJ Bailey. Induction of KLF4 in basal keratinocytes blocks the proliferation–differentiation switch and initiates squamous epithelial dysplasia. Oncogene. (2005) 24:1491–500. 10.1038/sj.onc.1208307 15674344 PMC1361530

[63] Villodre ES Kipper FC Pereira MB Lenz G. Roles of OCT4 in tumorigenesis, cancer therapy resistance and prognosis. Cancer Treat Rev. (2016) 51:1–9. 10.1016/j.ctrv.2016.10.003 27788386

[64] Bradshaw AR Wickremesekera AC Brasch HD Chibnall AM Davis PF. Glioblastoma multiforme cancer stem cells express components of the renin–angiotensin system. Front Surg. (2016) 3:51. 10.3389/fsurg.2016.00051 27730123 PMC5037176

[65] Itinteang T Dunne JC Chibnall AM Brasch HD Davis PF Tan ST. Cancer stem cells in moderately differentiated oral tongue squamous cell carcinoma express components of the renin-angiotensin system. J Clin Pathol. (2016) 69:942–5. 10.1136/jclinpath-2016-203736 27371611 PMC5050289

[66] Featherston T Yu HH Dunne JC Chibnall AM Brasch HD Davis PF. Cancer stem cells in moderately differentiated buccal mucosal squamous cell carcinoma express components of the renin–angiotensin system. Front Surg. (2016) 3:52. 10.3389/fsurg.2016.00052 27730124 PMC5037224

[67] Ram RS Brasch HD Dunne JC Davis PF Tan ST Itinteang T. Cancer stem cells in moderately differentiated lip squamous cell carcinoma express components of the renin–angiotensin system. Front Surg. (2017) 4:30. 10.3389/fsurg.2017.00030 28634582 PMC5459876

[68] Nallaiah S Lee VMY Brasch HD de Jongh J van Schaijik B Marsh R. Cancer stem cells within moderately differentiated head and neck cutaneous squamous cell carcinoma express components of the renin-angiotensin system. J Plast Reconstr Aesthet Surg. (2019) 72:1484–93. 10.1016/j.bjps.2018.11.013 30528285

[69] Matsushita K Wu Y Okamoto Y Pratt RE Dzau VJ. Local renin angiotensin expression regulates human mesenchymal stem cell differentiation to adipocytes. Hypertension. (2006) 48:1095–102. 10.1161/01.HYP.0000248211.82232.a7 17060512

[70] Rodgers K Xiong S Steer R DiZerega G. Effect of angiotensin II on hematopoietic progenitor cell proliferation. Stem Cells. (2000) 18:287–94. 10.1634/stemcells.18-4-287 10924095

