# Supplementary material for: Cancer Stem Cell Subpopulations Are Present Within Metastatic Head and Neck Cutaneous Squamous Cell Carcinoma
Source: Front Oncol. 2020 Jul 30;10:1091. doi: 10.3389/fonc.2020.01091 (PMC7406827; doi:10.3389/fonc.2020.01091)
Supplement: Supplementary file 1 [file Data_Sheet_1.docx]

Supplementary Materials


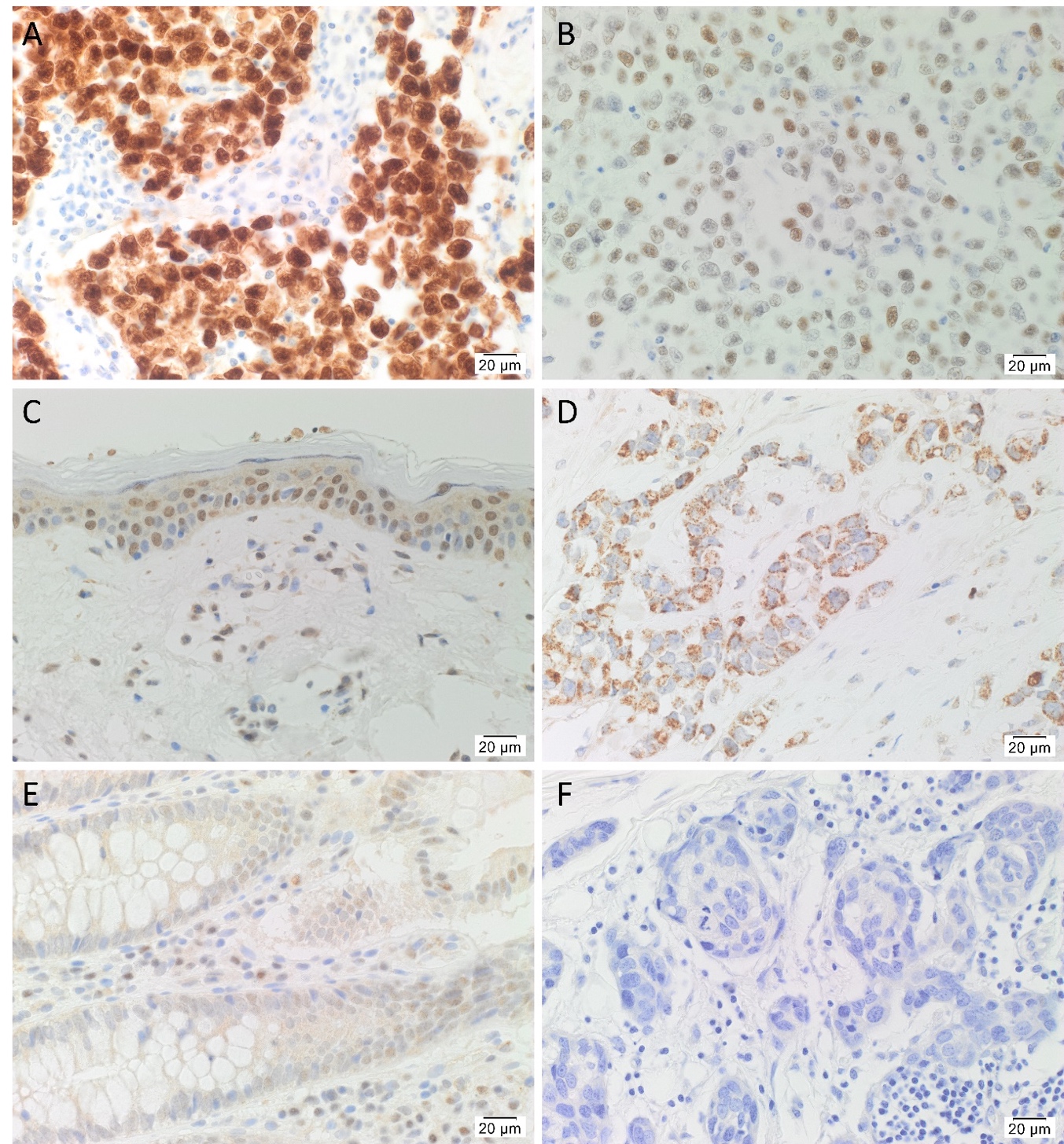


**Supplementary Figure 1.** Positive controls of immunohistochemical-staining of human tissues: seminoma for OCT4 (A, brown) and NANOG (B, brown), skin for SOX2 (C, brown), breast carcinoma for KLF4 (D, brown) and normal colon for c-MYC (E, brown). Specificity of staining was confirmed on a section of metastatic cutaneous squamous cell carcinoma using a matched isotype control for both mouse and rabbit primary antibodies (F, brown). Nuclei were counter-stained with hematoxylin (A-F, blue). Original magnification: 400x.


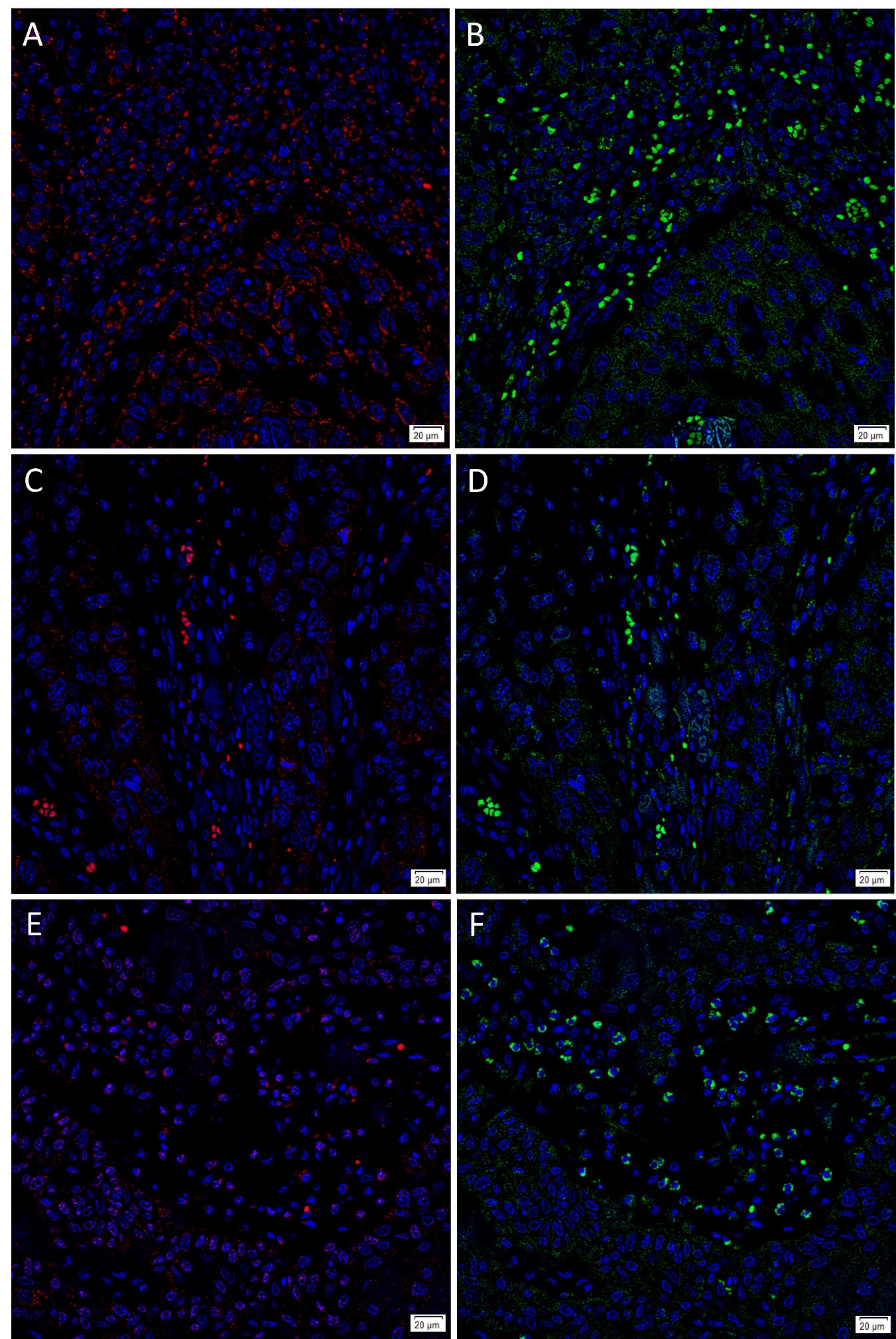


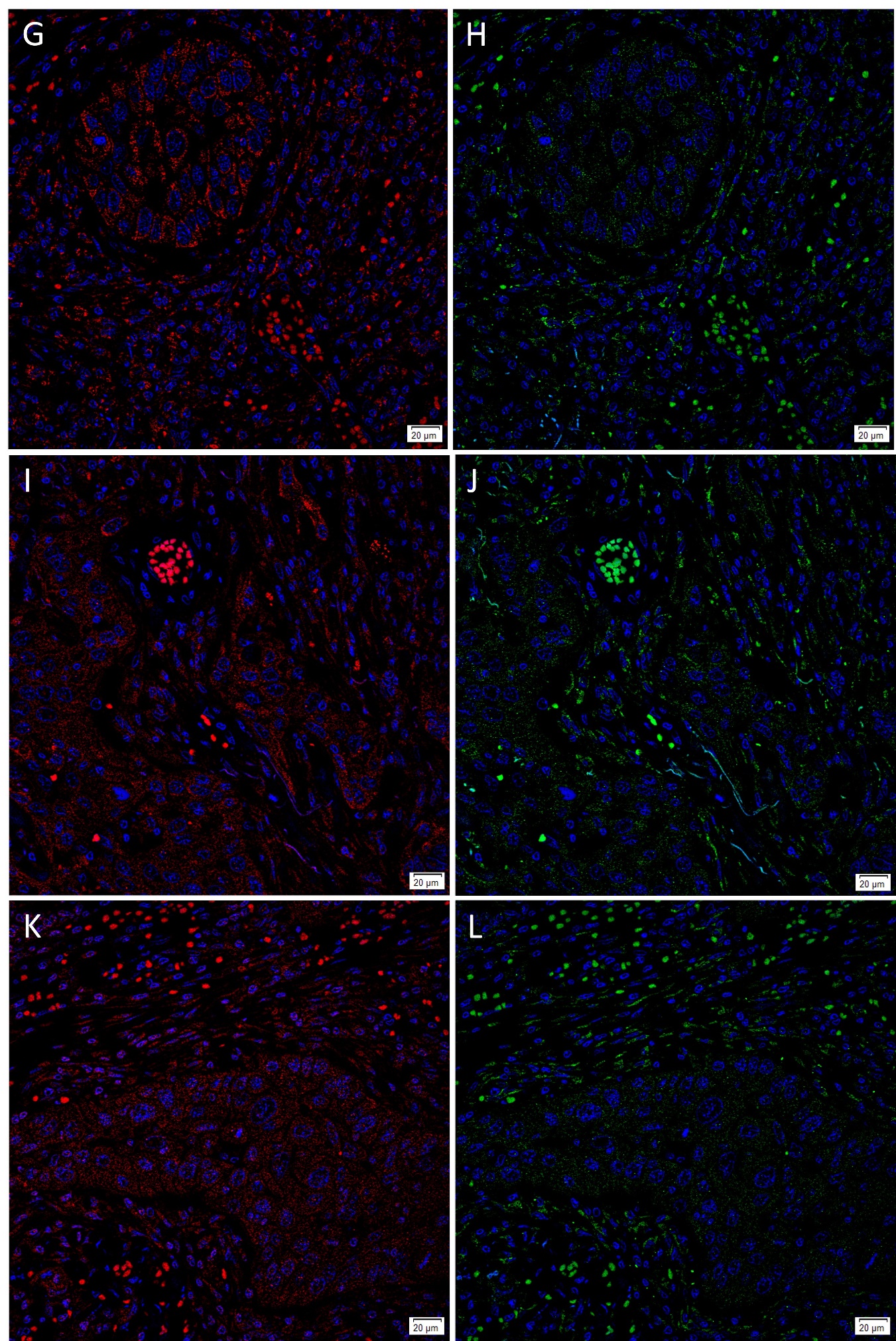


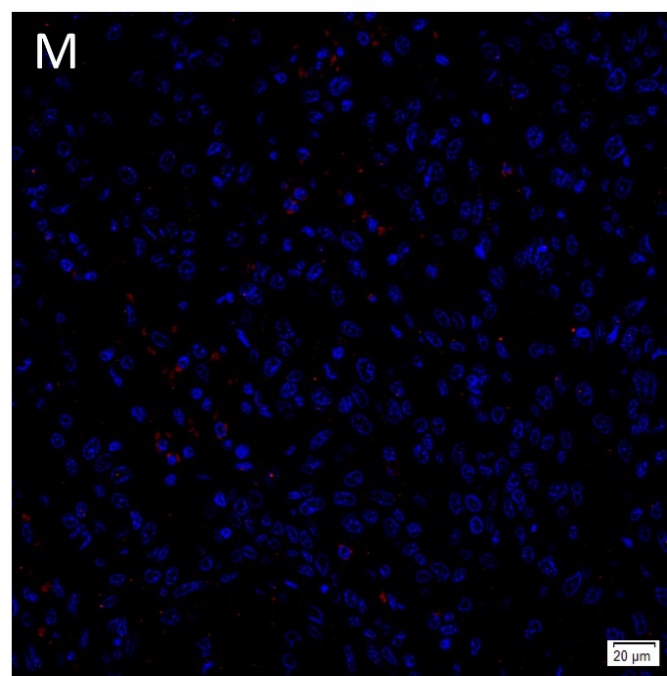
**Supplementary Figure 2.** Individual immunofluorescence stains of metastatic cutaneous head and neck squamous cell carcinoma (mHNcSCC) sections shown in Figure 2, demonstrating expression of KLF4 (A, red) and c-MYC (B, green); NANOG (C, red) and c-MYC (D, green); and SOX2 (E, red) and c-MYC (F, green); KLF4 (G, red) and OCT4 (H), green); and NANOG (I, red] and OCT4 (J, green) and SOX2 (K, red) and OCT4 (L, green). A negative control confirming the specificity of the fluorescent secondary antibodies is demonstrated on a section of mHNcSCC tissue sample (M). Cell nuclei were counter-stained with 4′,6′-diamidino-2-phenylindole (A-M, blue). Scale bars: 20µm.


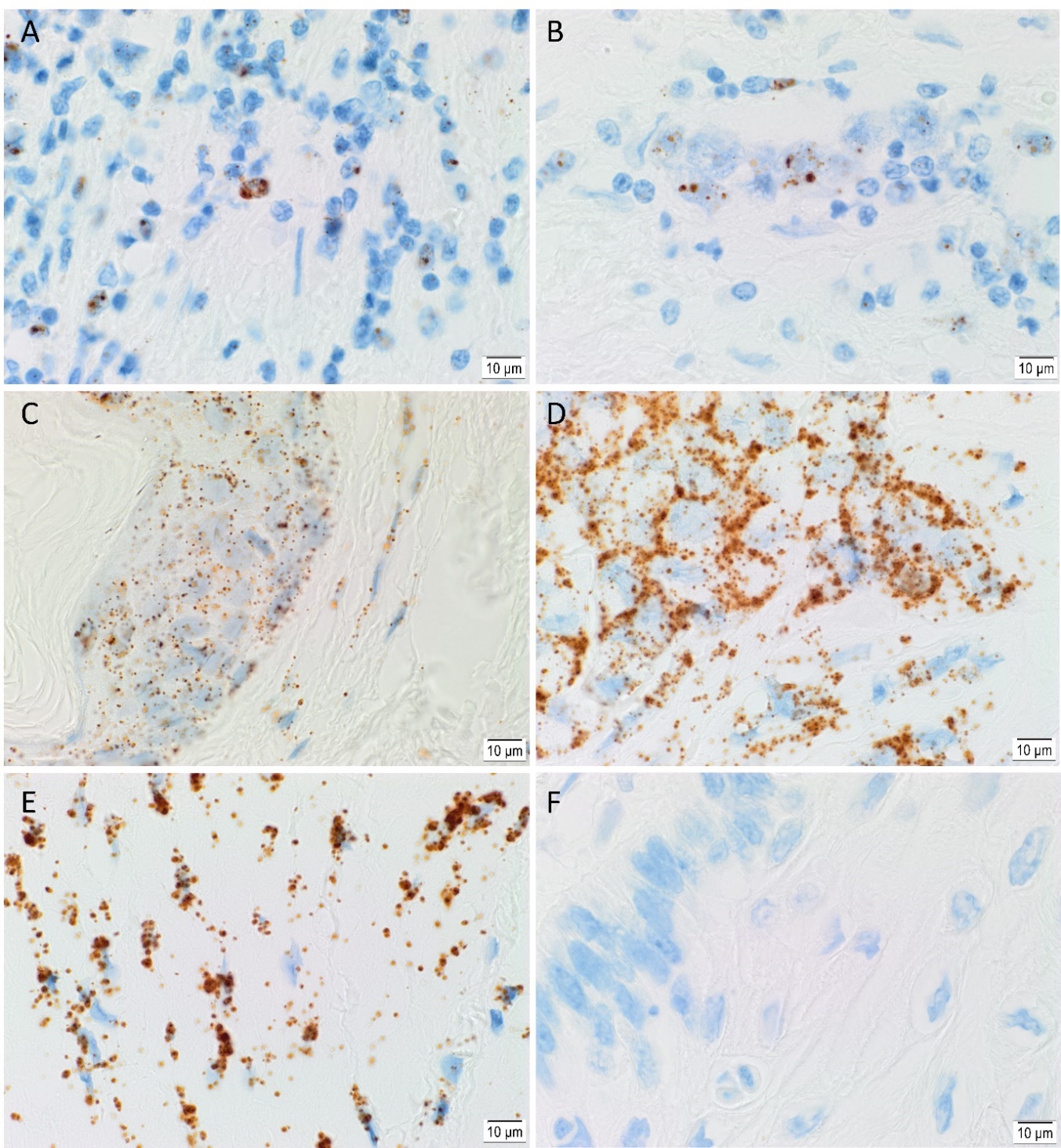


**Supplementary Figure 3.** Representative images of *in situ* hybridization stained slides of positive controls. These were demonstrated on sections of human tissues as follows: seminoma for OCT4 (A), and NANOG (B), skin for SOX2 (C), breast carcinoma for KLF4 (D), and colon for c-MYC (E); Negative control (F) demonstrated on sections of metastatic head and neck cutaneous squamous cell carcinoma tissue sample confirms specificity of secondary antibody. Original magnification: 1000x.


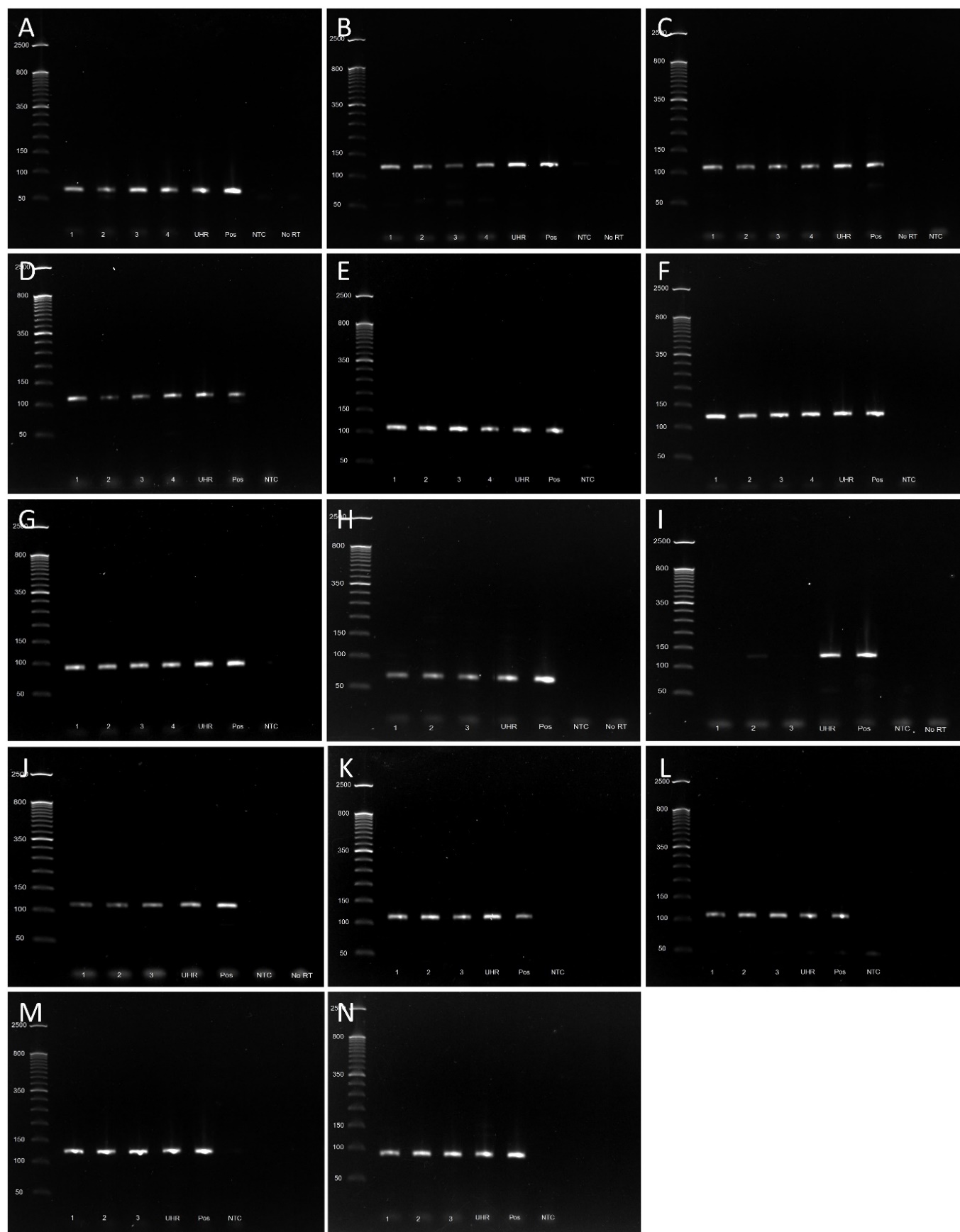


**Supplementary Figure 4.** Reverse transcription quantitative polymerase chain reaction (RT-qPCR) amplification products from four snap-frozen metastatic head and neck cutaneous squamous cell carcinoma (mHNcSCC) tissue samples (A-G) and three mHNcSCC-derived primary cell lines (H-N) were checked using agarose gel electrophoresis. Four mHNcSCC tissue replicate qPCR amplification products were probed for OCT4 (**A**, 64bp), SOX2 (**B**, 109bp), NANOG (**C**, 91bp), KLF4 (**D**, 110bp) and c-MYC (**E**, 107bp). Three mHNcSCC-derived primary cell lines qPCR amplification products were probed for OCT4 (**H**, 64bp), SOX2 (**I**, 109bp), NANOG (**J**, 91bp), KLF4 (**K**, 110bp) and c-MYC (**L**, 107bp). The housekeepers, GAPDH (122bp) and PUM1 (63bp), from mHNcSCC tissue samples (**F**) and (**G)** and cell lines (**M**) and (**N**) were also checked. Ladder refers to the DNA marker in base pairs (bp); lanes 1-5 refer to the respective tissue/cell samples; Pos, positive control (NTERA2 cell lines); NTC, no template control (RNase-free water) to confirm no contamination; No RT, Reverse transcriptase negative control for primers that may detect genomic DNA.


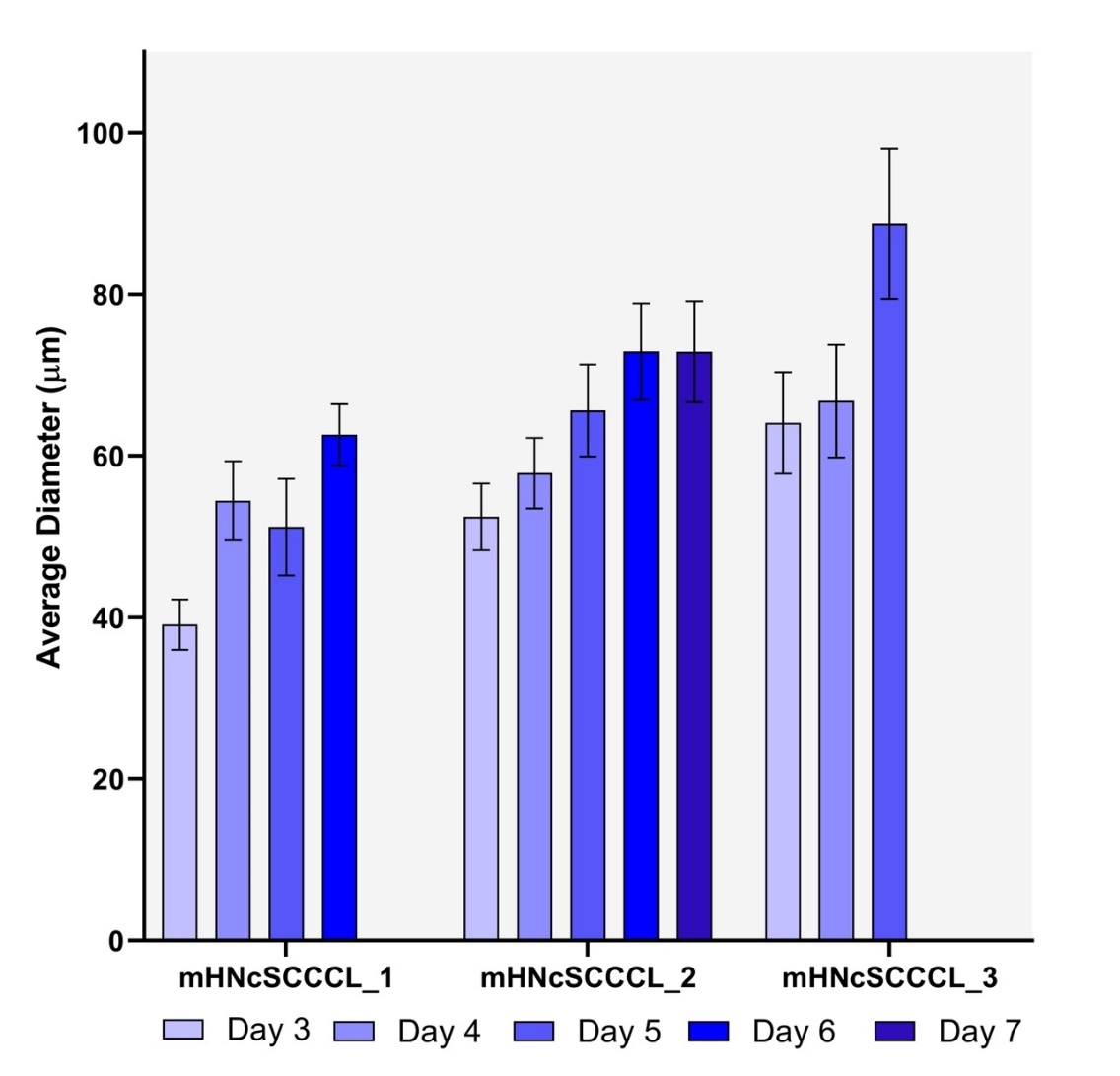


**Supplementary Figure 5.** A graph showing the mean diameter of tumorspheres derived from metastatic head and neck cutaneous squamous cell carcinoma-derived primary cell lines (mHNCSCCCL) over time averaging 62.6µm, 72.9µm and 88.7µm, respectively for the three cell lines. Error bars are 95% confidence intervals.
